# Improved efficacy of therapeutic HPV DNA vaccine using intramuscular injection with electroporation compared to conventional needle and needle-free jet injector methods

**DOI:** 10.1186/s13578-024-01338-x

**Published:** 2024-12-25

**Authors:** Shiwen Peng, Darrell Fan, Hsin-Fang Tu, Michelle Cheng, Rebecca C. Arend, Kimberly Levinson, Julia Tao, Richard B. S. Roden, Chien-Fu Hung, T.-C. Wu

**Affiliations:** 1 Department of Pathology, Johns Hopkins School of Medicine, CRB II Room 307, 1550 Orleans St, Baltimore, MD USA; 2 Department of Oncology, Johns Hopkins School of Medicine, CRB II Room 307, 1550 Orleans St, Baltimore, MD USA; 3 Department of Obstetrics and Gynecology, Johns Hopkins University, CRB II Room 307, 1550 Orleans St, Baltimore, MD USA; 4 Department of Obstetrics and Gynecology, University of Alabama at Birmingham, O’Neal Comprehensive Cancer Center, Birmingham, AL USA

**Keywords:** Human papillomavirus, HPV16, HPV18, E6, E7, DNA vaccine, HPV-associated cancers, Needle-free jet injector, Electroporation

## Abstract

**Background:**

We have previously developed a candidate therapeutic HPV DNA vaccine (pBI-11) encoding mycobacteria heat shock protein 70 linked to HPV16/18 E6/E7 proteins for the control of advanced HPV-associated oropharyngeal cancer (NCT05799144). While naked DNA vaccines are readily produced, stable, and well tolerated, their potency is limited by the delivery efficiency. Here we compared three different IM delivery strategies, including intramuscular (IM) injection, either with a needle alone or with electroporation at the injection site, and a needle-free injection system (NFIS), for their ability to elicit gene expression and to improve the potency of pBI-11 DNA vaccine.

**Results:**

We found that electroporation after IM injection significantly increases gene expression from a luciferase-encoding DNA construct compared to IM injection alone or NFIS. We also showed that single administration of pBI-11 DNA via electroporation-mediated delivery generates the greatest increase in HPV antigen-specific CD8 + T cell-mediated immune responses, resulting in the most potent antitumor effect compared to the other two methods. We further compared the response to three repeat immunizations via each of these different methods. We found that electroporation-mediated delivery of pBI-11 DNA generates the greatest HPV antigen-specific CD8 + T cell immune responses and therapeutic antitumor effects compared to the other two methods. Monitoring of mouse behaviors and body weight, and necropsy indicated that electroporation-mediated delivery of clinical grade pBI-11 DNA vaccine was well-tolerated and presented no evident local or systemic toxicity.

**Conclusions:**

These findings provide rationale for clinical testing of pBI-11 DNA vaccine delivered by electroporation for the control of HPV16/18-associated infections and/or cancers.

**Supplementary Information:**

The online version contains supplementary material available at 10.1186/s13578-024-01338-x.

## Introduction

Human papillomavirus (HPV) is a prevalent viral infection that remains a global health burden with a high incidence rate each year despite the development of prophylactic vaccines [1, 2]. While it is typical for low-risk HPV infections to regress overtime, over a dozen types of high-risk HPV (HR-HPV) contribute to the development of anal, cervical, oropharyngeal, penile, vaginal, and vulvar cancer [3–6]. The HR-HPV genotypes 16 and 18 represent the majority of variants in HPV-associated cancers, accounting for approximately 70–90% of all cervical and oropharyngeal cancers [4, 7–9].

The identification of HPV as the primary and necessary etiological factor presents an opportunity to employ virus-specific therapeutic strategies for the treatment of HPV-associated malignancies. The licensed prophylactic vaccines comprised of empty virus-like particles from the major viral capsid antigen L1 of several HPV genotypes, which induces production of high titers of protective antibodies [10, 11]. However, these prophylactic vaccines demonstrate no therapeutic efficacy against pre-existing HPV infections and associated diseases [12–15]. In addition, the efficacy of current surgical, chemo- and radiotherapies are limited, especially for patients with recurrent or metastatic HPV-associated cancers [16–20]. A promising new approach to improve patient outcomes is the utilization of immunotherapeutic HPV vaccines to augment antigen-specific T cell immunity against HPV early proteins, potentially in combination with immune checkpoint blockade [21].

Unlike the other HPV antigens, the E6 and E7 viral oncoproteins are always upregulated in HPV-associated cancers, serving as key drivers of tumorigenesis and maintenance of the transformed phenotype [22, 23]. These oncogenic proteins are exclusively expressed by HPV-infected cells and not in normal cells, rendering them ideal targets for tumor-specific immune cell recognition [24]. Furthermore, these foreign antigens are not subjected to central immune tolerance as they are non-self [25]. These characteristics provide a biological rationale for testing HPV immunotherapeutics targeting HPV E6/E7.

Among the various approaches to deliver HPV E6/E7 for vaccination, naked DNA plasmid expression constructs have considerable potential. We developed the pBI-11 DNA vaccine, which expresses a fusion protein linking the mycobacterium tuberculosis heat shock protein 70 (HSP70) with HPV16 E6/E7 and HPV18 E6/E7 proteins [26, 27]. HSP70 has demonstrated binding to CD91 on dendritic cells [28], so this may target the linked HPV-specific antigens to dendritic cells and greatly enhances the cross presentation of HPV16 E6/E7 and HPV18 E6/E7 through major histocompatibility complex (MHC) class I molecules [29]. Notably, pBI-11 DNA administration generates HPV E6/E7-specific CD8 + responses and demonstrates a strong therapeutic effect in controlling HPV16 + TC-1 tumors in mice [27]. The GMP grade pBI-11 DNA vaccine is currently undergoing testing in patients with advanced HPV + head and neck cancer (NCT05799144).

The potency of naked DNA vaccination is limited by the efficiency of their delivery in vivo [26, 30]. Strategies such as augmenting needle-based IM injection with in vivo electroporation (EP), and needle-free injection system (NFIS) have shown promise in the clinical setting [31, 32]. Conventional needle-based gene delivery often elicits minimal immunogenicity due to low intracellular transfection rate [33]. A promising strategy to improve transfection rates is to administer EP at the injection site following administration of the DNA [34]. This technique utilizes electric fields to momentarily destabilize the cellular membrane for normally impermeable macromolecules to enter the cell [35, 36]. At appropriate field strengths dependent on tissue and plasmid types, this technique can produce between 100 and 2000-fold increases in gene expression while imposing minimal damage on the tissues [35–38]. EP, including with the Papivax Biotech Inc. (PBI) TriGrid® device, has been safely utilized in many clinical studies for diverse indications, although none have been licensed to date [39–46].

Another strategy is the utilization of needle-free injection system (NFIS) technology via a high-pressure stream of liquid to puncture the skin and deliver the DNA intradermally and IM [47–49]. Their ease of use, absence of needles, high patient compliance, and cost-effectiveness makes NFIS particularly attractive for mass vaccination initiatives in resource-limited settings [48, 50–52]. Although several IM devices have been tested (e.g. Pharmajet Stratis® device, BioJector2000), the Pharmajet Tropis® biojector system demonstrated successful intradermal gene delivery and immune response of a prophylactic SARS-CoV-2 vaccine ZyCoV-D, licensed for clinical use in India [53].

In the current study, we compared the HPV antigen-specific CD8 + T cell-mediated immune response, as well as antitumor effect generated via IM injection with needle alone or followed by electroporation (with a PBI TriGrid® device modified for use in mice), to administration with needle-free jet device (with a Pharmajet Tropis® modified for use in mice). We found that IM administration of pBI-11 DNA vaccine via electroporation demonstrated the greatest HPV antigen-specific CD8 + T cell immune responses, antitumor effect, and overall survival among these delivery methods. Furthermore, we demonstrated that IM administration of GMP grade pBI-11 DNA vaccine via electroporation is well-tolerated without evident local or systemic toxicity in mice.

## Materials and methods

### Mice

6–8 weeks old female C57BL/6 mice were purchased from Charles River Laboratories (Wilmington, MA) and housed at the animal facility of Johns Hopkins University School of Medicine (Baltimore, MD) under specific-pathogen free conditions. All experimental procedures were approved by the Johns Hopkins University Animal Care and Use Committee and follow the Guide for the Care and Use of Laboratory Animals 8th ed (NRC 2011). We adhered to the guideline that the maximum tumor size for tumor-bearing mice should not exceed 2 cm in diameter.

### Peptides, antibodies and other reagents

Peptides of HPV16 E7aa49-57, RAHYNIVTF and HPV18 E6aa67-75, KCIDFYSRI, were synthesized by GenScript (Piscataway, NJ) at a purity of ≥ 80%. FITC, PE or APC/Fire™ 750 PE-conjugated anti-mouse CD8a (clone 53.6.7), FITC-conjugated anti-mouse IFN-γ (clone XMG1.2), and Brilliant Violet 650™ conjugated anti-mouse CD3 (clone 17A2) antibodies were purchased from Biolegend (San Diego, CA). PE-conjugated, HPV16 E7aa49-57 peptide loaded H-2D^b^ tetramers were purchased from MBL International Corporation (Woburn, MA). Purified rat anti-mouse CD16/32 (clone 2.4G2) were purchased from Bio X Cell (West Lebanon, NH). Bovine serum albumin (BSA) was purchased from Sigma (St. Louis, MI). Luciferin substrate was obtained from Xenogen Corp. (Alameda, CA). GolgiPlug and 7-AAD were purchased from BD Pharmingen (San Diego, CA). Intracellular fixation & permeabilization buffer set were purchased from eBioscience (San Diego, CA). Insulin Syringes with BD Ultra-Fine needle were purchased from BD Bioscience (NDC/HRI# 08290–3284-38, Franklin Lakes, NJ). A needle-free jet injector device was purchased from PharmaJet (Golden, CO). This needle-free injection system (NFIS) is customized for use in mice (modified for 0.05 mL delivery) with the C503-32 Tropis Syringe (loaded via a C503-42 Tropis Filling Adapter). ABTS solution was purchased from Roche (Indianapolis, IN). HRP-linked anti-mouse IgG whole antibody (from sheep) was purchased from GPR (GE Healthcare UK Limited, Little Chalfont Buckinghamshire, UK).

### Cell lines

HPV16 E6 and E7-expressing murine tumor cell line TC-1 was established as previously described[54]. These cell lines were maintained in RPMI medium supplemented with 2 mM glutamine, 1 mM sodium pyruvate, 100 IU/mL penicillin, 100 μg/mL streptomycin, non-essential amino acid and 10% fetal bovine serum (FBS) (Gibco, Grand Island, NY).

### DNA plasmid vaccine construct

Firefly luciferase-expressing DNA plasmid, pcDNA3-luciferase, was described previously[55]. pcDNA3-luciferase DNA was prepared using an endotoxin-free kit (QIAGEN, Valencia, CA). DNA vaccine pBI-11, encoding E6 and E7 proteins from both HPV16 and HPV18, has been described previously[27]. pBI-11 was prepared and vialed under cGMP by Waisman Biomanufacturing (Madison, WI) at 3 mg/mL in PBS (lot no. PPV-pBI-11-FP-001).

### Detection of luciferase expression

Female C57BL/6 mice (n = 5/group) were anesthetized with ketamine and injected with 4 μg of pcDNA3-luciferase plasmid via different devices. The first group underwent intramuscular (IM) injection with a conventional needle on the right tibialis anterior muscle. The second group underwent IM injection with needle-free Tropis biojector on the right tibialis anterior muscle. The third group underwent IM injection with conventional needle followed by electroporation (EP) using Intramuscular TriGrid Delivery System (TDS-IM, Papivax Biotech Inc. [PBI] USA, San Diego, CA) on the right tibialis anterior muscle. The luminescence was monitored by imaging with IVIS Lumina III In Vivo Imaging System (PerkinElmer, Inc., Waltham, MA).

### Vaccination Strategy

To compare the immunogenicity of pBI-11 administered through IM injection with different devices, DNA was diluted from cGMP grade stock with PBS to concentrations of 0.5 μg/μl and 1.25 μg/μl respectively. For IM injection with conventional needle or needle-free jet injector, 25 μg/50 μl of pBI-11 DNA was injected on the right tibialis anterior muscle. For IM injection with conventional needle followed by EP, 25 μg/20 μl of pBI-11 DNA was injected on the right tibialis anterior muscle. For the experiment with prime/boost vaccination, the mice were boosted twice with 7-day interval using the same regimen.

### Tetramer staining

For tetramer staining, mouse PBMCs or splenocytes were incubated with purified anti-mouse CD16/32 first. Then, it was stained with BV-650-conjugated anti-mouse CD3, APC/Fire™ 750-conjugated anti-mouse CD8a and PE-conjugated HPV16 E7aa49–57 peptide loaded H-2D^b^ tetramer at 4 °C for 1 h. Alternatively, mouse PBMCs were incubated with purified anti-mouse CD16/32 first, and then stained with FITC-conjugated anti-mouse CD8a and PE-conjugated HPV16/E7aa49–57 peptide loaded H-2D^b^ tetramer at 4 °C for 1 h. The cells were stained with 7-AAD before flow cytometry analysis to exclude dead cells. The cells were acquired with CytoFlex S (Beckman Coulter, Brea, CA) or Attune NxT (Thermal Fisher Scientific, Waltham, MA) flow cytometer and analyzed with FlowJo software from BD Biosciences (Franklin Lakes, NJ).

### Intracellular cytokine staining

To detect HPV16 E7 or HPV18 E6-specific CD8^+^ T cell responses by IFN-γ intracellular staining, splenocytes from vaccinated or naïve C57BL/6 mice were stimulated with either HPV16 E7aa49-57 or HPV18 E6aa67-75 peptide (1 µg/mL) in the presence of GolgiPlug (1 µL/mL) at 37 °C overnight. The stimulated splenocytes were then stained with PE-conjugated anti-mouse CD8a antibody. Cells were fixed, permeabilized, and further stained for intracellular IFN-γ with FITC-conjugated anti-mouse IFN-γ antibody. Flow cytometry analysis was performed using the CytoFlex S or Attune flow cytometer and analyzed with FlowJo software from BD Biosciences.

### Detection of HPV16 E7-specific antibody response by an enzyme-linked immunoabsorbent assay (ELISA)

To detect HPV16 E7-specific antibody response after pBI-11 DNA vaccination, naïve female C57BL/6 mice were vaccinated with 25 µg of pBI-11 DNA intramuscularly with either conventional needle, needle-free jet injector (Tropis device), or conventional needle followed by electroporation. The mice were boosted twice in 7-day intervals using the same regimen. 7 days after the last vaccination, sera from naïve and vaccinated mice were collected and HPV16 E7-specific antibody response was detected by ELISA as described before [56]. The optical density (OD) value was read with xMark microplate spectrophotometer (BioRad, Hercules, CA) ELISA reader at 405 nm with a reference wavelength of 490 nm.

### In vivo tumor prevention experiment

To compare the long-term antitumor immunity elicited by pBI-11 DNA vaccine, naïve female C57BL/6 mice (5 mice/group) were given single vaccination of 25 µg of pBI-11 DNA intramuscularly with either conventional needle, or conventional needle followed by electroporation, or NFIS (modified Tropis device). 30 days after vaccination, the mice were challenged with 5 × 10^5^ TC-1 cells subcutaneously. Tumor growth was monitored twice a week by palpation and digital caliper measurement. Tumor volume was calculated using the formula: [largest diameter × (perpendicular diameter)^2^] × 3.14/6. To record the survival, either natural death or a tumor diameter greater than 2 cm leading to death was counted as death.

### In vivo tumor treatment experiment

To compare the therapeutic effect of pBI-11 DNA vaccine administered through different devices, female C57BL/6 mice (n = 8/group) were implanted with 2 × 10^5^ TC-1 tumor cells subcutaneously. The tumor-bearing mice were divided into 4 groups. The first group did not receive any vaccination (untreated). The second group was vaccinated with 25 μg/50 μl of pBI-11 DNA through IM injection using a conventional needle (IM with needle). The third group was vaccinated with 25 μg/50 μl of pBI-11 DNA through IM injection using needle-free jet injector (IM with needle-free). The last group was vaccinated with 25 μg/20 μl of pBI-11 DNA through IM injection with conventional needle followed by electroporation (IM with EP). Mice were vaccinated with pBI-11 DNA vaccine on day 4 (when TC-1 tumor volume reached around 50 mm^3^). These mice were boosted twice in 3 days intervals using the same regimen. Tumor growth was monitored twice a week by palpation and digital caliper measurement. Tumor volume was calculated using the formula [largest diameter × (perpendicular diameter)^2^] × 3.14/6. To record survival, either natural death or a tumor diameter greater than 2 cm was counted as death.

### Assessment for impact of vaccination on behavior and physiological status of mice

To assess the impact of pBI-11 DNA vaccination through IM injection with needle followed by electroporation, the health of naïve female C57BL/6 mice was monitored by measurements of behaviors, body weight, and injection site irritation throughout the experiment and up to 1-week post-final vaccination per the JHU Animal Pathobiology and Phenotyping manual. 7 days after the last vaccination, complete blood counting (CBC) was performed at the JHU Animal Pathobiology and Phenotyping Core. A comprehensive biochemical analysis was performed by IDEXX BioAnalytics (West Sacramento, CA). In addition, necropsy was performed 1 week after the last vaccination (day 21), key organ weights were measured, and histology was examined by a board-certified pathologist.

### Measurement of DNA concentration between test and control pBI-11 DNA

Analysis of DNA concentration on the test and control samples was performed as specified in Additional file 2: Table S7. The concentration of the DNA was obtained through absorbance measures at 260 nm using the NanoDrop one spectrophotometer (Thermo Fisher Scientific, MA, US).

### Endotoxin quantification between test and control pBI-11 DNA

Analysis of endotoxin levels in test and control samples was performed as specified in Additional file 2: Table S7. Briefly, endotoxin levels in the test and control samples were measured via Kinetic-QCL™ Kinetic Chromogent LAL Assay (Catalog #: 50-650U, Lonza, Basel, CH).

### pH measurement between test and control pBI-11 DNA

Briefly the pH of DNA plasmids was determined using the SevenDirect™ SD50 pH/Ion Meter with InLab Micro electrode (Mettler Toledo, CH).

### DNA isoform separation and quantification by HPLC between test and control pBI-11 DNA

Analysis of DNA isoform was performed in test and control samples as specified in Additional file 2: Table S7. Briefly, HPLC was performed using 1260 II Infinity Systems (Agilent, USA) with TSKgel DNA-NPR (2.5 µm) column (Merck, DE) for separation and quantification. Buffer A (20 mM Tris, 0.5 M NaCl, pH 9.0) and Buffer B (20 mM Tris, 1 M NaCl, pH 9.0) were used in the mobile phase with the following parameters: flow rate of 1.0 mL/min, detection wavelength of 254 nm, column temperature of 25 °C, and injection volume of 30 µL. Before sample injection, the column was equilibrated with Buffer A for at least 15–20 min, and Buffer A was used as the blank sample to check the baseline. Once the baseline was stable, plasmid DNA and the pUC18 reference standard (Thermo Fisher Scientific, MA, US) was injected into the system to analyze the percentage of isoforms.

### Potency/transcriptional activity between test and control pBI-11 DNA

Analysis of the potency of test and control samples was assessed to document HPV16 E7-containing antigen expression of the appropriate molecular weight as specified in Additional file 2: Table S7. Briefly, HEK-293 cells were transfected with 2 µg (μg) of the DNA construct. After 48 h of transfection, cell lysates were collected for western blot analysis. The proteins were separated on a Precast Tris–HCl protein gel (GenScript, NJ, US) and then transferred onto a nitrocellulose membrane (Bio-Rad® Laboratories, CA, US). After blocking, the membrane is hybridized with HPV16 E7 antibody in 0.5 μg/ml at 4 °C overnight (8C9, Thermo Fisher Scientific, MA, US) to assess the expression level of HPV16 E7 antigen. Antibody binding was detected using a peroxidase conjugated goat anti-mouse secondary antibody in 0.8 μg/ml at RT for 1 h (Thermo Fisher Scientific, MA, US). Visualization is performed using chemiluminescence (ECL + detection kit, Amersham™, NJ, US), and images were captured with a ChemiDoc-It 815 Image System (Analytik Jena, CA, US).

### Statistical analysis

Data were expressed as means ± standard deviations or Box and Whisker plots showing 10–90 percentiles. Statistical analysis was performed using Prism software (version 10.2.1). Comparisons between individual data point was analyzed by 2-tailed Student’s *t* test (Mann–Whitney test). Survival outcomes were displayed using the Kaplan–Meier curves and compared by the log-rank test. *p-*values ≤ 0.05 were considered significant (*, p < 0.05; **, p < 0.01; ***, p < 0.001).

## Results

### Mice vaccinated with pBI-11 DNA delivered by IM injection followed by electroporation demonstrated increased HPV antigen-specific CD8 + T cell responses compared to IM with needle or with NFIS

**Fig. 1:**
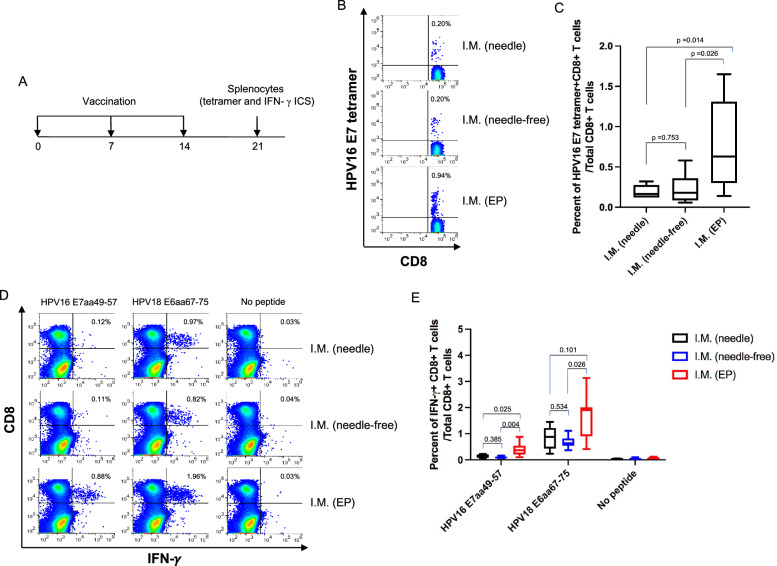
Comparison of HPV antigen-specific CD8 + T cell responses after IM vaccination with pBI-11 DNA using either needle, NFIS, or needle followed by electroporation.** A** Schema of the experiment. Female C57BL/6 mice (6 ~ 7 mice/group) were used. Mice of the first group were vaccinated with 25 μg of pBI-11 DNA/mouse through intramuscular (IM) injection on the right tibialis anterior muscle with needle. Mice of the second group were vaccinated with 25 μg of pBI-11 DNA/mouse through IM injection on the right tibialis anterior muscle with the Tropis NFIS. Mice of the third group were vaccinated with 25 μg of pBI-11 DNA/mouse through IM injection on the right tibialis anterior muscle with a needle followed by electroporation. All groups were boosted twice with the same regimen in 1-week intervals. 7 days after the last vaccination, splenocytes were harvested from all mice and HPV antigen-specific CD8 + T cells were characterized by E7 peptide-loaded H-2D^b^ tetramer, as well as intracellular IFN-$\gamma$ cytokine staining followed by flow cytometry analysis using the methods as described in the Materials & Methods section **B** Representative flow cytometry images of HPV16 E7aa49-57 peptide-specific CD8 + T cells detected by HPV16 E7 tetramer staining assay. **C** Box plot summary of HPV16 E7aa49-57 peptide-specific CD8 + T cells detected by HPV16 E7 tetramer staining assay after pBI-11 DNA vaccination. **D** Representative flow cytometry images of HPV16 E7aa49-57 and HPV18 E6aa67-75 peptide-specific CD8 + T cells detected by IFN-γ intracellular staining assay. **E** Box plot summary of HPV16 E7aa49-57 and HPV18 E6aa67-75 peptide-specific CD8 + T cells detected by IFN-γ intracellular staining assay after pBI-11 DNA vaccination

We evaluated the HPV antigen-specific CD8 + T cell responses among mice vaccinated with pBI-11 DNA vaccine through 3 different delivery methods: IM with needle, IM with EP (PBI’s TriGrid electroporation device modified for use in mice), or NFIS (Pharmajet’s Tropis device modified for use in mice). C57BL/6 mice were vaccinated 3 times at one week intervals, and their splenocytes were harvested 7 days after the last vaccination (Fig. 1A). Our results revealed that pBI-11 vaccination by IM with needle and IM with NFIS showed comparable levels of circulating HPV16 E7-specific CD8 + T cells using HPV16 E7 peptide-loaded tetramer staining (Fig. 1B, C). In addition, IM with EP showed significantly higher levels of circulating HPV16 E7-specific CD8 + T cells than that of IM with needle and IM with NFIS using HPV16 E7 peptide-loaded tetramer staining (Fig. 1B, C). Furthermore, intracellular IFN-$\gamma$ cytokine staining followed by flow cytometry analysis revealed that mice vaccinated by IM with EP showed significant increase in HPV16 E7aa49-57 and HPV18 E6aa67-75 peptide-specific CD8 + T cell responses compared to mice vaccinated by IM with needle or with NFIS (Fig. 1D, E). However, mice vaccinated by NFIS did not generate significantly different HPV16 E7aa49-57 and HPV18 E6aa67-75 peptide-specific CD8 + T cell responses as compared to mice vaccinated by needle only (Fig. 1D, E). Although there were no significant differences observed in the HPV16 E7-specific antibody response among the three delivery methods, EP-mediated needle delivery elicited greater HPV16 E7-specific antibody responses (Additional file 1: Figure S1). Altogether, our data indicate that mice vaccinated with pBI-11 by IM with EP showed significantly greater HPV antigen-specific CD8 + T cell responses compared to the other two methods.

### IM injection followed by electroporation can enhance the expression of the encoded gene of the DNA construct compared to intramuscular injection with needle or NFIS

**Fig. 2:**
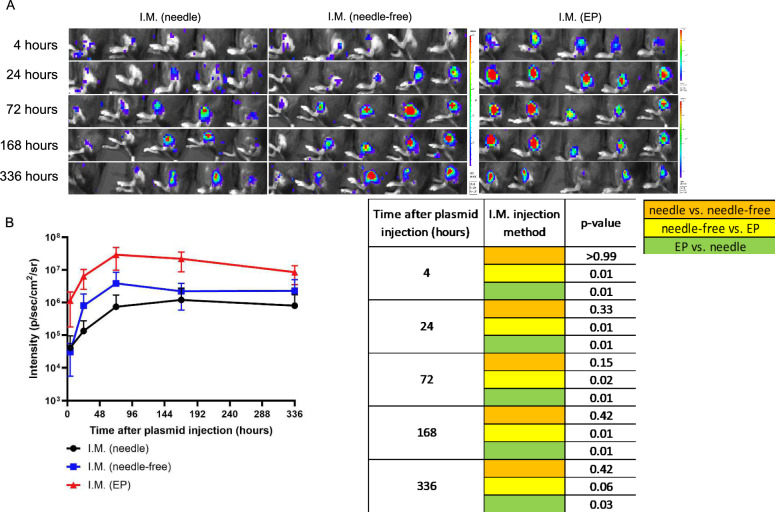
Comparison of luciferase expression after IM injection of pcDNA3-Luciferase with either needle, NFIS, or needle followed by EP. Female C57BL/6 mice (5 mice/group) were used. Mice of the first group were injected with 4 μg in 50 μL/mouse of pcDNA3-luciferase through IM injection with needle on the right tibialis anterior muscle. Mice of the second group were injected with 4 μg in 50 μL/mouse of pcDNA3-luciferase through NFIS (modified Tropis, PharmaJet) on the right tibialis anterior muscle. Mice of the third group were injected with 4 μg in 20 μL/mouse of pcDNA3-luciferase through IM injection with needle followed by electroporation. The luciferase expression was monitored by IVIS Lumina III imaging system. **A** Bioluminescence image of luciferase expression after pcDNA3-luciferase plasmid injection. **B** Line graph summary of luciferase expression monitored by bioluminescence imaging after pcDNA3-luciferase plasmid injection. Corresponding table shows the *P* values (Mann–Whitney test) of the differences in luciferase expression between each of the injection delivery approaches over time

Female C57BL/6 mice were administered pcDNA3-Luciferase via IM with needle, or IM with EP, or NFIS (modified Tropis device). The luciferase expression was monitored 4, 24, 72, 168, and 336 h post vaccination (Fig. 2A). Among the three delivery methods, IM with EP demonstrated significantly stronger intensity in luciferase expression compared to IM with needle or NFIS at the first 4 time points (4, 24, 72, and 168th hour) (Fig. 2B). Conversely, IM with needle compared to NFIS generated similar levels of luciferase expression at all 5 time points (Fig. 2B). This data suggests that conventional needle delivery with EP represents the best approach to enhance gene expression from naked plasmid DNA among these methods.

### One vaccination with pBI-11 DNA via IM injection followed by electroporation generated the highest HPV16 E7 and HPV18 E6-specific CD8 + T cell immune responses overtime among the three delivery methods

**Fig. 3:**
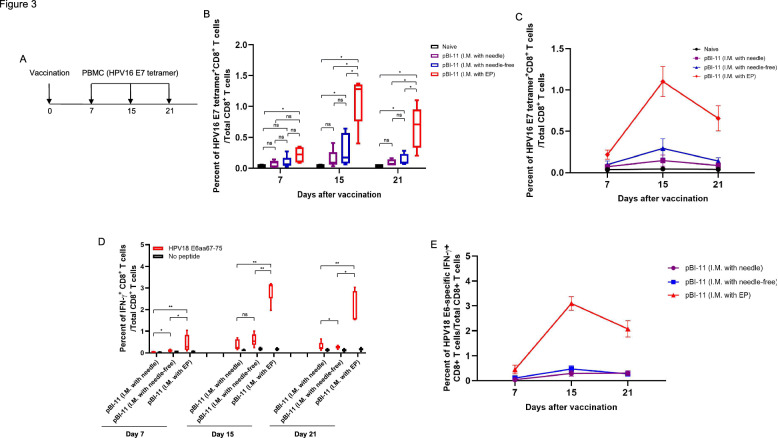
Kinetics of HPV antigen-specific CD8 + T cell responses after single IM pBI-11 DNA vaccination with either needle, NFIS, or needle followed by EP. **A** Schema of the experiment. Female C57BL/6 mice (5 mice/group) were used. Mice of the first group were vaccinated with 25 μg of pBI-11 DNA/mouse through intramuscular (IM) injection on the right tibialis anterior muscle with needle. Mice of the second group were vaccinated with 25 μg of pBI-11 DNA/mouse through IM injection on the right tibialis anterior muscle with NFIS (Tropis). Mice of the third group were vaccinated with 25 μg of pBI-11 DNA/mouse through IM injection on the right tibialis anterior muscle with a needle followed by electroporation. PBMCs were collected from naïve and vaccinated mice at the indicated time (Day 7, 15, 21). After red blood cell lysis, some PBMCs were stained with FITC-conjugated anti-mouse CD8a and PE-conjugated HPV16 E7aa49-57 peptide loaded H-2D^b^ tetramer. The remaining PBMCs were stimulated with HPV18 E6aa67-75 peptide (1 μg/ml) in the presence of GolgiPlug (1 μl/ml) overnight. The cells were characterized for HPV18 E6-specific CD8 + T cells using intracellular IFN-$\gamma$ cytokine staining followed by flow cytometry analysis using the methods as described in Materials & Methods section. **B** Box plot summary of flow cytometry analysis of HPV16 E7aa49-57 peptide-specific CD8 + T cells detected by HPV16 E7-tetramer staining assay. **C** Line graph summary of HPV16 E7aa49-57 peptide-specific CD8 + T cells detected by HPV16 E7-tetramer staining assay. **D** Box plot summary of flow cytometry analysis of HPV18 E6aa67-75 peptide-specific CD8 + T cells detected by IFN-γ intracellular staining assay. **E** Line graph summary of HPV18 E6aa67-75 peptide-specific CD8 + T cells detected by IFN-γ intracellular staining assay after pBI-11 DNA vaccination. ∗ , p ≤ 0.05; ∗  ∗ , p ≤ 0.01; ns, not significant

We evaluated the kinetics of HPV16 E7 and HPV18 E6-specific CD8 + T cell responses from mice vaccinated with pBI-11 DNA vaccine through 3 different delivery methods: IM with needle, NFIS, and IM with EP. C57BL/6 mice were vaccinated once before PBMCs were collected a total of three times at approximately one week intervals after the last vaccination (Fig. 3A). On Day 7, there were no significant differences in the generated HPV16 E7-specific CD8 + T cell responses from the three different methods (Fig. 3B, C). Yet, on Day 15 and 21, it is clear that IM with EP generated significantly greater levels of HPV16 E7-specific CD8 + T cell responses compared to either IM alone or NFIS (Fig. 3B, C). This suggests that delivery via IM injection and EP better increases and maintains higher levels of HPV16 E7-specific CD8 + T cell responses than either IM alone or NFIS. Similar results were observed in HPV18 E6-specific IFN-$\gamma$+ CD8 + T cell responses. On Day 7, there were significant differences in the generated HPV18 E6-specific IFN-$\gamma$+ CD8 + T cell responses by IM with EP compared to the other two methods (Fig. 3D, E). By Day 15 and 21, IM with EP also generated significantly greater levels of HPV18 E6-specific IFN-$\gamma$+ CD8 + T cell responses compared to the other two methods (Fig. 3D, E). Altogether, our data indicates that IM with EP represents the best approach to generate HPV antigen-specific CD8 + T cell immune responses among all the tested methods.

### A single vaccination with pBI-11 DNA vaccine via IM injection followed by electroporation demonstrated the strongest protective antitumor immunity against TC-1 tumor challenge

**Fig. 4:**
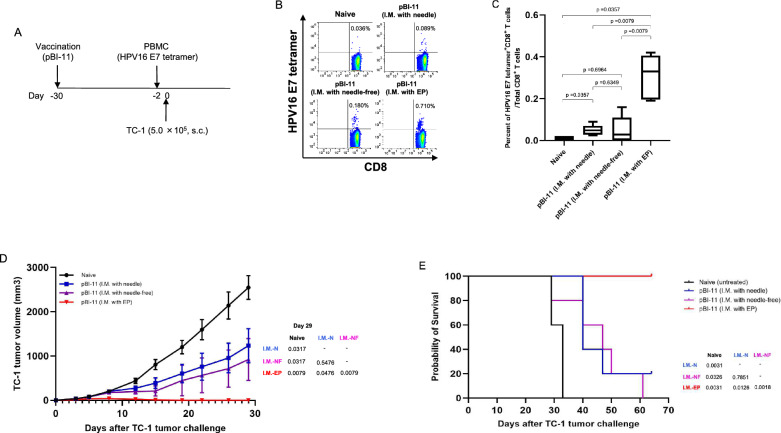
Comparison of preventative anti-tumor immunity of single IM pBI-11 DNA vaccination with either needle, NFIS, or needle followed by EP. **A** Schema of the experiment. 30 days prior to TC-1 tumor implantation, female C57BL/6 mice (5 mice/group) were vaccinated with 25 μg of pBI-11 DNA/mouse through intramuscular (IM) injection on the right tibialis anterior muscle with either conventional needle (IM with needle), a modified Tropis NFIS (needle-free), or with a conventional needle followed by EP. At 2 days prior to TC-1 tumor implantation, PBMCs were prepared from the naïve and vaccinated mice to detect HPV16 E7aa49-57 peptide loaded H-2D^b^ tetramer + CD8a + T cells. On Day 0, these naïve and vaccinated mice were subcutaneously challenged with 5.0 × 10^5^ TC-1 cells on the right flank of the abdomen. Growth of TC-1 tumor was monitored twice a week by palpation and digital caliper measurement. Tumor volume was calculated using the formula: [largest diameter × (perpendicular diameter)^2^] × 3.14/6. To record the survival of tumor-bearing mice, either natural death or a tumor diameter greater than 2 cm were counted as death. **B** Representative flow cytometry image of HPV16 E7-specific CD8 + T cells in peripheral blood using HPV16 E7 tetramer staining assay. **C** Box plot summary of HPV16 E7-specific CD8 + T cells in peripheral blood detected by HPV16 E7 tetramer staining. **D** Summary of the TC-1 tumor growth curve for each treatment group. **E.** Kaplan Meier analysis of the survival of TC-1 tumor-bearing mice. *IM-N* IM with needle, *IM-NF* IM with needle-free, *IM-EP* I.M with EP. Displayed log-rank p-values indicate differences in survival between groups

Next, we evaluated the protective effect of pBI-11 DNA vaccine using the three methods against the TC-1 tumor challenge. One month before TC-1 tumor implantation, mice were vaccinated with pBI-11 DNA vaccine using IM with needle alone, NFIS, or IM with EP. PBMCs were collected to quantify the level of HPV16 E7-specific CD8 + T cells two days before the tumor challenge begins (Fig. 4A). As expected, pBI-11 DNA administered via IM with EP generated significantly higher levels of HPV16 E7-specific CD8 + T cells compared to administration via either IM with needle alone or NFIS. Meanwhile, there were no significant differences in the levels of HPV16 E7-specific CD8 + T cells generated by IM with needle and NFIS (Fig. 4B, C). Furthermore, we observed that vaccinated mice using IM with needle and NFIS slowed tumor growth compared to control mice. When comparing all three delivery methods, IM with EP led to the best tumor control with no tumor growth. (Fig. 4D). It is noteworthy that there is high variability in tumor volumes present in each group (Additional File 1: Figure S2). The potent antitumor effect generated by IM with EP vaccination significantly prolonged overall survival as compared to mice from the other groups (Fig. 4E). Taken together, our data suggests that pBI-11 DNA vaccination via IM with EP generated the strongest preventative antitumor effect among all methods tested against TC-1 tumor challenge.

### Tumor-bearing mice treated with pBI-11 DNA via IM injection followed by electroporation demonstrated the best therapeutic antitumor effect among all the three delivery methods

**Fig. 5:**
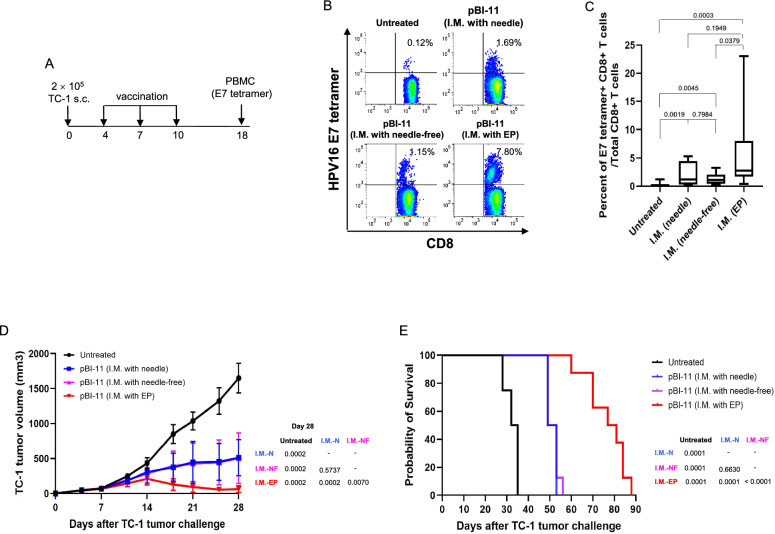
Comparison of therapeutic anti-tumor immunity in TC-1 tumor-bearing C57BL/6 mice after pBI-11 DNA vaccination with either needle, NFIS, or needle followed by EP.** A** Schema of the experiment. Female C57BL/6 mice (11- to 12-week-old, 8 mice/group) were injected with 2 × 10^5^ TC-1 cells subcutaneously on Day 0. On Day 4, the mice were divided into 4 groups. The first group was used as the untreated control. The second group was vaccinated with 25 µg of pBI-11 DNA/mouse through intramuscular (IM) injection with needle. The third group was vaccinated with a total of 25 µg of pBI-11 DNA/mouse through IM injection with customized needle-free Tropis system. The fourth group was vaccinated with a total of 25 µg of pBI-11 DNA/mouse through IM injection with needle followed by electroporation. All groups were boosted with their corresponding regimen for 3 times at 3-day intervals. Tumor growth was monitored twice a week by palpation and digital caliper measurement. Tumor volume was calculated using the formula: [largest diameter × (perpendicular diameter)^2^] × 3.14/6. To record the survival of tumor-bearing mice, either natural death or a tumor diameter greater than 2 cm was counted as death. **B** Representative flow cytometry image of HPV16 E7-specific CD8 + T cells in peripheral blood using HPV16 E7 tetramer staining assay. **C** Box plot summary of HPV16 E7-specific CD8 + T cells in peripheral blood detected by HPV16 E7 tetramer staining assay. **D** Summary of the TC-1 tumor growth curve for each treatment group. **E** Kaplan Meier analysis of the survival of TC-1 tumor-bearing mice. *IM-N* IM with needle, *IM-NF* IM with needle-free, *IM-EP* I.M with EP. Displayed log-rank p-values indicate differences in survival between groups

For clinical translation purposes, we performed the standard prime/boost/boost vaccination with pBI-11 DNA. Using this protocol, we evaluated the anti-tumor immunity of mice vaccinated three times with pBI-11 using one of the three different methods. C57BL/6 mice were inoculated with TC-1 tumor and vaccinated on days 4, 7, and 10, and then PBMCs were isolated and stained with HPV16 E7 tetramer 8 days after the final vaccination (Fig. 5A**)**. Our results revealed that IM with needle and NFIS produced similar levels of HPV16 E7-specific CD8 + T cells, but both were higher than in untreated mice (Fig. 5B, C). Furthermore, we found that IM with EP generated significantly higher HPV16 E7-specific CD8 + T cell immune response compared to NFIS **(**Fig. 5B, C**).** We also showed that IM with EP tended to generate higher HPV16 E7-specific CD8 + T cell immune response compared to IM with needle alone, although significance was not reached **(**Fig. 5B, C**).** All treated mice had significantly slower tumor growth than that of untreated mice. While there were no significant differences of tumor growth between mice treated by IM with needle and with NFIS, mice treated via injection IM and EP generated the best therapeutic antitumor effect with the slowest tumor growth among all groups (Fig. 5D). This tumor growth paralleled findings in the survival curve. First, all vaccinated mice, regardless of delivery methods, demonstrated significantly longer survival than untreated mice. Of the three delivery methods, IM with EP generated the best overall survival (Fig. 5E). Overall, our results indicated that tumor-bearing mice treated with pBI-11 DNA delivered via IM with EP generates the greatest HPV antigen-specific CD8 + T cell responses, the slowest tumor growth, and the longest overall survival.

### pBI-11 DNA vaccination delivered by IM injection with needle followed by EP is well tolerated

Although IM with EP is the best strategy to boost the immunogenicity of pBI-11 DNA administration, it remains unclear whether this delivery method can be tolerated by mice. To investigate the tolerability, we divided C57BL/6 mice into 2 groups of 5 mice. The first group was left unvaccinated. The second group was vaccinated with pBI-11 DNA through IM injection with EP on the right tibialis anterior muscle. The mice were boosted twice with the same regimen at a 1-week interval, and mice were sacrificed a week after the final vaccination (Additional file 1: Figure S3A). The health of each mouse was closely monitored by observing their body weight, behaviors, and injection site reaction. All vaccinated mice were healthy for the entire experiment and demonstrated typical behavioral phenotypes for all assessments (Additional file 2: Table S1). The injection sites of vaccinated mice were observed at 2- and 24-h post vaccination and reveal no eschar (Additional file 2: Table S2) or edema formation (Additional file 2: Table S3) in all groups. Moreover, mouse body weight in all vaccination groups remained consistent throughout the experimental duration (Additional file 1: Figure S3B). One week following the final vaccination, we conducted necropsy and performed key organ weight measurements, complete blood count, clinical chemistry analysis, and histological studies. The mean organ weights were comparable in both groups (Additional file 1: Figure S4). Complete blood count measurements were unremarkable and similar in both groups (Additional file 2: Table S4), and biochemistry readouts showed no significant differences between the groups (Additional file 2: Table S5). Histological examination of key organs showed results within normal limits for mice in both groups (Additional file 2: Table S6). Altogether, this indicates that IM with EP of pBI-11 DNA vaccine is safe and well tolerated by mice with no local or systemic toxicity.

### Injection through the clinical grade TriGrid® Electroporation device does not change the inherent qualities of the clinical grade pBI-11 DNA

For clinical translation, it is intended that the pBI-11 DNA vaccine will be administered via the clinical grade TriGrid® Electroporation device. In order to demonstrate the suitability of this administration method, pBI-11 samples were subjected to the preparation and administration conditions associated with the TriGrid® Electroporation device and then collected for analysis. Negative control samples comprised unconditioned pBI-11 samples from the same manufacturing lot. Following completion of the simulated administration procedures, the pBI-11 test samples were evaluated for appearance, pH, endotoxin levels, DNA concentration, DNA isoform. All test samples met the pBI-11 specifications for visual appearance (clear colorless solution with no detectable particulate or foreign matter, pH [7], endotoxin content ($\le$ 10 EU/mg), DNA concentration (3.0 $\pm$ 0.3 mg/mL), supercoiled plasmid DNA isoform content ($\ge$ 80% supercoiled), and potency (positive for HPV16 E7 expression) with no notable differences compared to the unconditioned negative control samples. Additional file 2: Table S7 summarizes the results of these comparisons. Specifically, the initial tested pBI-11 DNA showed differences in pH, but this is influenced by exposure to dry ice which decreases the pH through formation of carbonic acid [57]. Subsequent testing not subjected to dry ice exposure demonstrated consistency in pH with control pBI-11 DNA. In addition, the chromatograms of tested pBI-11 DNA showed that the device conditioning did not result in any change in the pBI-11 DNA supercoiled plasmid DNA isoform content (Additional file 1: Figure S5). Quantification of Western blot results also demonstrated that the tested pBI-11 DNA expressed a HPV16 E7-containing protein of consistent molecular weight and expression level as compared to the control pBI-11 DNA when transfected into HEK-293 cells (Additional file 1: Figure S6). Altogether, our multifaceted approaches indicated that the inherent quality of the clinical grade pBI-11 DNA does not change following injection through the clinical grade TriGrid® Electroporation device.

## Discussion

We tested different delivery approaches, namely IM injection with conventional needle, NFIS (modified Tropis device), and IM injection followed by EP. Most notably, the electroporation-mediated pBI-11 DNA vaccination generated the highest increase in HPV16 E7 and HPV18 E6-specific T cell levels and best antitumor effects. The observed antitumor effects were demonstrated in both the preventative and treatment settings. Moreover, we observed that IM with EP was safe and well tolerated by mice with no local or systemic toxicity. These data suggests that IM with EP is a potentially desirable option for clinical translation.

Our study is one of the first to compare the therapeutic efficacy generated by electroporation and NFIS. In our previous publication, we identified NFIS as a preferred choice based on its potential to facilitate a therapeutic anti-tumor effect without the use of a needle [58]. This study expands options for delivery of naked plasmid DNA vaccines by demonstrating that electroporation-mediated delivery generates an even greater preventative and therapeutic effect against tumor burden when compared to both IM with needle and NFIS, although it does require use of multiple needles and is transiently painful **(**Figs. 4, 5**)**. Interestingly, several groups documented the effect of using electroporation and NFIS together [59–62]. For instance, Hallengard et al. showed that the combination of both a jet injector and an electroporation system can significantly raise the therapeutic effect of plasmid DNA vaccines [63]. Our approach demonstrates that electroporation alone is sufficient to increase therapeutic efficacy of DNA-based vaccines.

The use of EP to enhance vaccine potency has well-established biological rationale. EP not only increases transfection efficiency and thus gene expression of naked DNA as compared to IM injection only, it also activates both cellular and humoral immune responses by several mechanisms. First, EP generates local inflammation through greater secretion of IL-1 $\beta$ and TNF $\alpha$ and increases antibody levels [64–66]. In addition, this strategy promotes dendritic cell maturation, leading to greater antigen presentation and T-cell stimulatory abilities [67]. Furthermore, EP triggers an innate immune response by releasing damage-associated molecular pattern (DAMPs) molecules which, upon recognition by receptors such as toll-like receptors (TLRs) and NOD-like receptors (NLR), trigger an inflammatory response [68, 69]. Thus, EP can activate both innate and adaptive immunity as well as transfection efficiency to enhance vaccine potency.

One significant challenge with vaccines that encode multiple antigens is the phenomenon known as “immunodominance”. This effect occurs when the immune system preferentially targets the immunodominant antigen, resulting in a robust antigen-specific response against it, while responses to other antigens are suppressed [70, 71]. Consequently, vaccines designed to present multiple antigens may be limited to inducing strong immune responses against only a subset, and not all, encoded antigens. Our data corroborate this phenomenon, demonstrating that IM with needle and NFIS resulted in approximately 8 times greater HPV18 E6-specific CD8 + T cell responses compared to HPV16 E7-specific CD8 + T cell responses **(**Fig. 1D**)**. On the other hand, IM with EP generated only 2 times greater HPV18 E6-specific CD8 + T cell responses compared to HPV16 E7-specific CD8 + T cell responses **(**Fig. 1D**)**. In total, IM with EP generated approximately 8 times greater HPV16 E7-specific CD8 + T cell responses and 2 times greater HPV18 E6-specific CD8 + T cell responses compared to IM with needle and NFIS **(**Fig. 1D**)**. Taken together, these data suggests that electroporation can generate the greatest HPV16 E7 and HPV18 E6-specific CD8 + T cell responses and mitigate the suppression of the subdominant CTL epitope by the immunodominant CTL epitope in antigen presentation. Therefore, electroporation-mediated delivery appears to reduce the effects of immunodominance and achieve more consistent antigen-specific T cell responses against both HPV16 and HPV18 as compared with the two other delivery methods tested for pBI-11 DNA. The exact mechanism for this observed phenomenon is not clear. Angeletti et al. suggested that increasing local antigen concentration in tumor-draining lymph nodes could overcome immunodominance [72]. Building on this finding, we speculate that EP greatly enhances the expression of HPV antigens encoded by pBI-11, thereby improving their recognition by the immune system despite their subdominant nature.

In our current study, we focused on administration of pBI-11 DNA with different clinically-relevant delivery methods and identified that IM with EP as a promising strategy to enhance the vaccine potency and antitumor effects. Despite this conclusion, we remain curious whether the current approach is a superior delivery method compared to other routes such as ID administration with NFIS. Previously, we showed that the ID administration of pBI-11 DNA with NFIS is the best strategy to increase HPV antigen-specific T cell responses and enhance antitumor effects compared to IM with NFIS and IM with needle alone [58]. Therefore, it is important to further test whether IM with EP or ID with NFIS is the best delivery strategy for pBI-11 DNA.

In addition, we also tested for safety and toxicity including mice behaviors, body weight, organ weights, histology, biochemical tests, and complete blood count. We did not find any local or systemic toxicity (see Additional file 1: Figure S3, S4 and Additional file 2: Table S1-S6). However, mice were under anesthesia during administration for humane reasons, so pain was not measurable during delivery. Several clinical trials have also demonstrated that electroporation-mediated delivery presents no major concerns with toxicities or complications [39, 43, 44, 46, 73], although transient pain involving the use of 3 needles, one for injection and two as electrodes, were noted. Together with these trials, we provided further evidence for the potential of electroporation due to its ability to enhance DNA vaccine potency and feasibility for the vaccine recipient based upon prior clinical experience.

The remarkable success of the mRNA-based prophylactic SARS-COV-2 vaccines [74] and recent promising results with personalized neoantigen-based mRNA vaccines for cancer immunotherapy [75, 76] raise questions about the need for DNA-based vaccination. Indeed, there are also two ongoing clinical trials exploring the mRNA-based BNT113 vaccine that targets HPV16 E6 and E7 proteins in HPV16-associated cancers (NCT03418480 and NCT04534205). These products use lipid nanoparticles (LNPs) for delivery, which enhances mRNA stability and facilitates simple injection methods [77]. Alternatively, DNA-based vaccines are known to be much more stable than mRNA, and are readily manufactured at low cost in bacteria. This study supports the potential of DNA vaccination but suggest that they need to be delivered by more complex strategies like electroporation-mediated delivery. The need for the EP device suggests that this approach is more suited to therapeutic indications like cancer and chronic viral infection rather than use for prophylaxis in healthy patient cohorts. Also, EP is more painful than simple IM injection, albeit only transient, and requires access to and training in use of the device, as well as additional consumables. Furthermore, the use of an electroporation device has several limitations, including transient pain for the recipient. While the associated manufacturing and regulatory issues around using an electroporator contribute to both the cost and complexity for clinical delivery, EP does not require complex formation with nanoparticles, for example. NFIS also require consumables (although not a needle) and training, but are generally well tolerated by patients suggesting that they are appropriate for use in prophylaxis or less severe diseases. Nonetheless, our study shows that NFIS is less effective for delivery of pBI-11 DNA vaccine because IM with EP induces a greater therapeutic T cell response. Our current study serves as important foundation for future clinical translation of pBI-11 DNA vaccine against HPV16 and HPV18-associated diseases.

## Supplementary Information

Supplementary Material 1

Supplementary Material 2

## Data Availability

All data and materials are available upon request from the corresponding authors Chien-Fu Hung (chung2@jhmi.edu) or T.-C. Wu (wutc@jhmi.edu).

## Abbreviations

BSA: Bovine serum albumin
CBC: Complete blood count
CD: Cluster of differentiation
COVID-19: Coronavirus disease 2019
CTL: Cytotoxic lymphocytes
DAMP: Damage-associated molecular pattern
DNA: Deoxyribose nucleic acid
EP: Electroporation
FBS: Fetal bovine serum
GMP: Good manufacturing practice
HPLC: High performance liquid chromatography
HPV: Human papillomavirus
HR-HPV: High-risk human papillomavirus
HSP70: Heat shock protein 70
ID: Intradermal
IFN-g: Interferon-gamma
IM: Intramuscular
LNP: Lipid nanoparticles
MHC: Major histocompatibility complex
mRNA: Messenger ribonucleic acid
NFIS: Needle-free injection system
NLR: NOD-like receptors
OD: Optical density
PBMC: Peripheral blood mononuclear cells
SARS: Severe acute respiratory syndrome
TLR: Toll-like receptors

## References

[1] Bruni L, Albero G, Rowley J, Alemany L, Arbyn M, Giuliano AR, et al. Global and regional estimates of genital human papillomavirus prevalence among men: a systematic review and meta-analysis. Lancet Glob Health. 2023;11(9):e1345–62. 37591583 10.1016/S2214-109X(23)00305-4 PMC10447222

[2] Kombe Kombe AJ, Li B, Zahid A, Mengist HM, Bounda GA, Zhou Y, et al. Epidemiology and burden of human papillomavirus and related diseases, molecular pathogenesis, and vaccine evaluation. Front Public Health. 2020;8: 552028. 33553082 10.3389/fpubh.2020.552028 PMC7855977

[3] Ferrall L, Lin KY, Roden RBS, Hung CF, Wu TC. Cervical cancer immunotherapy: facts and hopes. Clin Cancer Res. 2021;27(18):4953–73. 33888488 10.1158/1078-0432.CCR-20-2833 PMC8448896

[4] Forman D, de Martel C, Lacey CJ, Soerjomataram I, Lortet-Tieulent J, Bruni L, et al. Global burden of human papillomavirus and related diseases. Vaccine. 2012;30(Suppl 5):F12-23. 23199955 10.1016/j.vaccine.2012.07.055

[5] Gillison ML, Chaturvedi AK, Anderson WF, Fakhry C. Epidemiology of human papillomavirus-positive head and neck squamous cell carcinoma. J Clin Oncol. 2015;33(29):3235–42. 26351338 10.1200/JCO.2015.61.6995 PMC4979086

[6] Oyouni AAA. Human papillomavirus in cancer: infection, disease transmission, and progress in vaccines. J Infect Public Health. 2023;16(4):626–31. 36868166 10.1016/j.jiph.2023.02.014

[7] Chaturvedi AK, Engels EA, Pfeiffer RM, Hernandez BY, Xiao W, Kim E, et al. Human papillomavirus and rising oropharyngeal cancer incidence in the United States. J Clin Oncol. 2011;29(32):4294–301. 21969503 10.1200/JCO.2011.36.4596 PMC3221528

[8] Lechner M, Liu J, Masterson L, Fenton TR. HPV-associated oropharyngeal cancer: epidemiology, molecular biology and clinical management. Nat Rev Clin Oncol. 2022;19(5):306–27. 35105976 10.1038/s41571-022-00603-7 PMC8805140

[9] Roman BR, Aragones A. Epidemiology and incidence of HPV-related cancers of the head and neck. J Surg Oncol. 2021;124(6):920–2. 34558067 10.1002/jso.26687 PMC8552291

[10] Harper DM, Vierthaler SL, Santee JA. Review of Gardasil. J Vaccines Vaccin. 2010. 10.4172/2157-7463.1000107. 23805398 10.4172/2157-7560.1000107 PMC3690661

[11] Monie A, Hung CF, Roden R, Wu TC. Cervarix: a vaccine for the prevention of HPV 16, 18-associated cervical cancer. Biologics. 2008;2(1):97–105. 19707432 PMC2727782

[12] Cuzick J. Gardasil 9 joins the fight against cervix cancer. Expert Rev Vaccines. 2015;14(8):1047–9. 26028344 10.1586/14760584.2015.1051470

[13] Einstein MH, Baron M, Levin MJ, Chatterjee A, Edwards RP, Zepp F, et al. Comparison of the immunogenicity and safety of Cervarix and Gardasil human papillomavirus (HPV) cervical cancer vaccines in healthy women aged 18–45 years. Hum Vaccin. 2009;5(10):705–19. 19684472 10.4161/hv.5.10.9518

[14] Joura EA, Giuliano AR, Iversen OE, Bouchard C, Mao C, Mehlsen J, et al. A 9-valent HPV vaccine against infection and intraepithelial neoplasia in women. N Engl J Med. 2015;372(8):711–23. 25693011 10.1056/NEJMoa1405044

[15] Schiller JT, Müller M. Next generation prophylactic human papillomavirus vaccines. Lancet Oncol. 2015;16(5):e217–25. 25943066 10.1016/S1470-2045(14)71179-9

[16] Ang KK, Harris J, Wheeler R, Weber R, Rosenthal DI, Nguyen-Tân PF, et al. Human papillomavirus and survival of patients with oropharyngeal cancer. N Engl J Med. 2010;363(1):24–35. 20530316 10.1056/NEJMoa0912217 PMC2943767

[17] Roof L, Yilmaz E. Immunotherapy in HPV-related oropharyngeal cancers. Curr Treat Options Oncol. 2023;24(3):170–83. 36719604 10.1007/s11864-023-01050-x PMC9887557

[18] Shamseddine AA, Burman B, Lee NY, Zamarin D, Riaz N. Tumor immunity and immunotherapy for HPV-related cancers. Cancer Discov. 2021;11(8):1896–912. 33990345 10.1158/2159-8290.CD-20-1760 PMC8338882

[19] Timbang MR, Sim MW, Bewley AF, Farwell DG, Mantravadi A, Moore MG. HPV-related oropharyngeal cancer: a review on burden of the disease and opportunities for prevention and early detection. Hum Vaccin Immunother. 2019;15(7–8):1920–8. 31050595 10.1080/21645515.2019.1600985 PMC6746516

[20] Ward G, Mehta V, Moore M. Morbidity, mortality and cost from HPV-related oropharyngeal cancer: Impact of 2-, 4- and 9-valent vaccines. Hum Vaccin Immunother. 2016;12(6):1343–7. 26566988 10.1080/21645515.2015.1095415 PMC4964634

[21] Yang A, Farmer E, Wu TC, Hung CF. Perspectives for therapeutic HPV vaccine development. J Biomed Sci. 2016;23(1):75. 27809842 10.1186/s12929-016-0293-9 PMC5096309

[22] Yang A, Jeang J, Cheng K, Cheng T, Yang B, Wu TC, et al. Current state in the development of candidate therapeutic HPV vaccines. Expert Rev Vaccines. 2016;15(8):989–1007. 26901118 10.1586/14760584.2016.1157477 PMC4977850

[23] Zur Hausen H. Papillomaviruses and cancer: from basic studies to clinical application. Nat Rev Cancer. 2002;2(5):342–50. 12044010 10.1038/nrc798

[24] Doorbar J, Egawa N, Griffin H, Kranjec C, Murakami I. Human papillomavirus molecular biology and disease association. Rev Med Virol. 2015;25(Suppl 1):2–23. 25752814 10.1002/rmv.1822 PMC5024016

[25] Brianti P, De Flammineis E, Mercuri SR. Review of HPV-related diseases and cancers. New Microbiol. 2017;40(2):80–5. 28368072

[26] Kutzler MA, Weiner DB. DNA vaccines: ready for prime time? Nat Rev Genet. 2008;9(10):776–88. 18781156 10.1038/nrg2432 PMC4317294

[27] Peng S, Ferrall L, Gaillard S, Wang C, Chi WY, Huang CH, et al. Development of DNA vaccine targeting E6 and E7 proteins of human papillomavirus 16 (HPV16) and HPV18 for immunotherapy in combination with recombinant vaccinia boost and PD-1 antibody. MBio. 2021. 10.1128/mBio.03224-20. 33468698 10.1128/mBio.03224-20 PMC7845631

[28] Zimmer C, Henics T. Surface binding and uptake of heat shock protein 70 by antigen-presenting cells require all 3 domains of the molecule. Cell Stress Chaperones. 2002;7(3):243–9. 12482200 10.1379/1466-1268(2002)007<0243:sbauoh>2.0.co;2 PMC514824

[29] Arnold-Schild D, Hanau D, Spehner D, Schmid C, Rammensee HG, de la Salle H, et al. Cutting edge: receptor-mediated endocytosis of heat shock proteins by professional antigen-presenting cells. J Immunol. 1999;162(7):3757–60. 10201889

[30] Tsen SW, Paik AH, Hung CF, Wu TC. Enhancing DNA vaccine potency by modifying the properties of antigen-presenting cells. Expert Rev Vaccines. 2007;6(2):227–39. 17408372 10.1586/14760584.6.2.227 PMC3190226

[31] Ledesma-Feliciano C, Chapman R, Hooper JW, Elma K, Zehrung D, Brennan MB, et al. Improved DNA vaccine delivery with needle-free injection systems. Vaccines (Basel). 2023;11(2):280. 36851159 10.3390/vaccines11020280 PMC9964240

[32] Luz J, Antunes F, Clavijo-Salomon MA, Signori E, Tessarollo NG, Strauss BE. Clinical applications and immunological aspects of electroporation-based therapies. Vaccines (Basel). 2021;9(7):727. 34358144 10.3390/vaccines9070727 PMC8310106

[33] Wang S, Zhang C, Zhang L, Li J, Huang Z, Lu S. The relative immunogenicity of DNA vaccines delivered by the intramuscular needle injection, electroporation and gene gun methods. Vaccine. 2008;26(17):2100–10. 18378365 10.1016/j.vaccine.2008.02.033 PMC2790191

[34] Hu MH, Fan D, Tu HF, Tsai YC, He L, Zhou Z, et al. Electroporation-mediated novel albumin-fused Flt3L DNA delivery promotes cDC1-associated anticancer immunity. Gene Ther. 2024. 10.1038/s41434-024-00497-3. 39472678 10.1038/s41434-024-00497-3

[35] Jaroszeski MJ, Gilbert R, Nicolau C, Heller R. In vivo gene delivery by electroporation. Adv Drug Deliv Rev. 1999;35(1):131–7. 10837694 10.1016/s0169-409x(98)00068-4

[36] Young JL, Dean DA. Electroporation-mediated gene delivery. Adv Genet. 2015;89:49–88. 25620008 10.1016/bs.adgen.2014.10.003 PMC6005385

[37] Jorritsma SHT, Gowans EJ, Grubor-Bauk B, Wijesundara DK. Delivery methods to increase cellular uptake and immunogenicity of DNA vaccines. Vaccine. 2016;34(46):5488–94. 27742218 10.1016/j.vaccine.2016.09.062

[38] Sachdev S, Potočnik T, Rems L, Miklavčič D. Revisiting the role of pulsed electric fields in overcoming the barriers to in vivo gene electrotransfer. Bioelectrochemistry. 2022;144: 107994. 34930678 10.1016/j.bioelechem.2021.107994

[39] Elizaga ML, Li SS, Kochar NK, Wilson GJ, Allen MA, Tieu HVN, et al. Safety and tolerability of HIV-1 multiantigen pDNA vaccine given with IL-12 plasmid DNA via electroporation, boosted with a recombinant vesicular stomatitis virus HIV Gag vaccine in healthy volunteers in a randomized, controlled clinical trial. PLoS ONE. 2018;13(9): e0202753. 30235286 10.1371/journal.pone.0202753 PMC6147413

[40] Hannaman D, Dupuy LC, Ellefsen B, Schmaljohn CS. A Phase 1 clinical trial of a DNA vaccine for Venezuelan equine encephalitis delivered by intramuscular or intradermal electroporation. Vaccine. 2016;34(31):3607–12. 27206386 10.1016/j.vaccine.2016.04.077

[41] Hooper J, Paolino KM, Mills K, Kwilas S, Josleyn M, Cohen M, et al. A Phase 2a Randomized, double-blind, dose-optimizing study to evaluate the immunogenicity and safety of a bivalent DNA vaccine for hemorrhagic fever with renal syndrome delivered by intramuscular electroporation. Vaccines (Basel). 2020;8(3). 10.3390/vaccines8030377 PMC7565952 32664486

[42] Hooper JW, Moon JE, Paolino KM, Newcomer R, McLain DE, Josleyn M, et al. A Phase 1 clinical trial of Hantaan virus and Puumala virus M-segment DNA vaccines for haemorrhagic fever with renal syndrome delivered by intramuscular electroporation. Clin Microbiol Infect. 2014;20(Suppl 5):110–7. 24447183 10.1111/1469-0691.12553

[43] Mpendo J, Mutua G, Nanvubya A, Anzala O, Nyombayire J, Karita E, et al. Acceptability and tolerability of repeated intramuscular electroporation of Multi-antigenic HIV (HIVMAG) DNA vaccine among healthy African participants in a phase 1 randomized controlled trial. PLoS ONE. 2020;15(5): e0233151. 32469893 10.1371/journal.pone.0233151 PMC7259687

[44] Mpendo J, Mutua G, Nyombayire J, Ingabire R, Nanvubya A, Anzala O, et al. A phase I double blind, placebo-controlled, randomized study of the safety and immunogenicity of electroporated HIV DNA with or without interleukin 12 in prime-boost combinations with an Ad35 HIV vaccine in healthy HIV-seronegative african adults. PLoS ONE. 2015;10(8): e0134287. 26252526 10.1371/journal.pone.0134287 PMC4529153

[45] Spearman P, Mulligan M, Anderson EJ, Shane AL, Stephens K, Gibson T, et al. A phase 1, randomized, controlled dose-escalation study of EP-1300 polyepitope DNA vaccine against *Plasmodium falciparum* malaria administered via electroporation. Vaccine. 2016;34(46):5571–8. 27697302 10.1016/j.vaccine.2016.09.041 PMC5075504

[46] Vasan S, Hurley A, Schlesinger SJ, Hannaman D, Gardiner DF, Dugin DP, et al. In vivo electroporation enhances the immunogenicity of an HIV-1 DNA vaccine candidate in healthy volunteers. PLoS ONE. 2011;6(5): e19252. 21603651 10.1371/journal.pone.0019252 PMC3095594

[47] Ravi AD, Sadhna D, Nagpaal D, Chawla L. Needle free injection technology: a complete insight. Int J Pharm Investig. 2015;5(4):192–9. 26682189 10.4103/2230-973X.167662 PMC4675000

[48] Yu C, Walter M. CADTH Rapid Response Reports. Needleless Injectors for the Administration of Vaccines: A Review of Clinical Effectiveness. Ottawa (ON): Canadian Agency for Drugs and Technologies in Health Copyright © 2020 Canadian Agency for Drugs and Technologies in Health.; 2020. 33296154

[49] Hillemanns P, Denecke A, Woelber L, Böhmer G, Jentschke M, Schjetne KW, et al. A therapeutic antigen-presenting cell-targeting DNA vaccine VB1016 in HPV16-positive high-grade cervical intraepithelial neoplasia: results from a phase I/IIa trial. Clin Cancer Res. 2022;28(22):4885–92. 36129459 10.1158/1078-0432.CCR-22-1927

[50] Bullo UF, Mehraj J, Raza SM, Rasool S, Ansari NN, Shaikh AA, et al. An experience of mass administration of fractional dose inactivated polio vaccine through intradermal needle-free injectors in Karachi, Sindh, Pakistan. BMC Public Health. 2021;21(1):44. 33407294 10.1186/s12889-020-10041-8 PMC7789602

[51] Singh B, Maharjan S, Sindurakar P, Cho KH, Choi YJ, Cho CS. Needle-Free Immunization with Chitosan-Based Systems. Int J Mol Sci. 2018;19(11):3639. 30463211 10.3390/ijms19113639 PMC6274840

[52] Yousafzai MT, Saleem AF, Mach O, Baig A, Sutter RW, Zaidi AKM. Feasibility of conducting intradermal vaccination campaign with inactivated poliovirus vaccine using Tropis intradermal needle free injection system, Karachi, Pakistan. Heliyon. 2017;3(8): e00395. 29333501 10.1016/j.heliyon.2017.e00395 PMC5750384

[53] Khobragade A, Bhate S, Ramaiah V, Deshpande S, Giri K, Phophle H, et al. Efficacy, safety, and immunogenicity of the DNA SARS-CoV-2 vaccine (ZyCoV-D): the interim efficacy results of a phase 3, randomised, double-blind, placebo-controlled study in India. Lancet. 2022;399(10332):1313–21. 35367003 10.1016/S0140-6736(22)00151-9 PMC8970574

[54] Lin KY, Guarnieri FG, Staveley-O’Carroll KF, Levitsky HI, August JT, Pardoll DM, et al. Treatment of established tumors with a novel vaccine that enhances major histocompatibility class II presentation of tumor antigen. Cancer Res. 1996;56(1):21–6. 8548765

[55] Best SR, Peng S, Juang CM, Hung CF, Hannaman D, Saunders JR, et al. Administration of HPV DNA vaccine via electroporation elicits the strongest CD8+ T cell immune responses compared to intramuscular injection and intradermal gene gun delivery. Vaccine. 2009;27(40):5450–9. 19622402 10.1016/j.vaccine.2009.07.005 PMC2745985

[56] Cheng WF, Hung CF, Hsu KF, Chai CY, He L, Ling M, et al. Enhancement of sindbis virus self-replicating RNA vaccine potency by targeting antigen to endosomal/lysosomal compartments. Hum Gene Ther. 2001;12(3):235–52. 11177561 10.1089/10430340150218387

[57] Murphy BM, Swarts S, Mueller BM, van der Geer P, Manning MC, Fitchmun MI. Protein instability following transport or storage on dry ice. Nat Methods. 2013;10(4):278–9. 23538862 10.1038/nmeth.2409

[58] Peng S, Tu HF, Cheng M, Hu MH, Tsai HL, Tsai YC, et al. Immune responses, therapeutic anti-tumor effects, and tolerability upon therapeutic HPV16/18 E6/E7 DNA vaccination via needle-free biojector. MBio. 2023;14(5):e0212123. 37791765 10.1128/mbio.02121-23 PMC10653862

[59] Inoue S, Mizoguchi I, Sonoda J, Sakamoto E, Katahira Y, Hasegawa H, et al. Induction of potent antitumor immunity by intradermal DNA injection using a novel needle-free pyro-drive jet injector. Cancer Sci. 2023;114(1):34–47. 36000926 10.1111/cas.15542 PMC9807518

[60] Shapiro JR, Hodgins B, Hendin HE, Patel A, Menassa K, Menassa C, et al. Needle-free delivery of influenza vaccine using the Med-Jet® H4 is efficient and elicits the same humoral and cellular responses as standard IM injection: a randomized trial. Vaccine. 2019;37(10):1332–9. 30709725 10.1016/j.vaccine.2019.01.039

[61] Simon JK, Carter M, Pasetti MF, Sztein MB, Kotloff KL, Weniger BG, et al. Safety, tolerability, and immunogenicity of inactivated trivalent seasonal influenza vaccine administered with a needle-free disposable-syringe jet injector. Vaccine. 2011;29(51):9544–50. 21986218 10.1016/j.vaccine.2011.09.097

[62] Lambracht-Washington D, Fu M, Wight-Carter M, Riegel M, Hynan LS, Rosenberg RN. DNA Aβ42 immunization via needle-less Jet injection in mice and rabbits as potential immunotherapy for Alzheimer’s disease. J Neurol Sci. 2023;446: 120564. 36731358 10.1016/j.jns.2023.120564

[63] Hallengärd D, Bråve A, Isaguliants M, Blomberg P, Enger J, Stout R, et al. A combination of intradermal jet-injection and electroporation overcomes in vivo dose restriction of DNA vaccines. Genet Vaccines Ther. 2012;10(1):5. 22873174 10.1186/1479-0556-10-5 PMC3532290

[64] Adam L, Tchitchek N, Todorova B, Rosenbaum P, Joly C, Poux C, et al. Innate Molecular and Cellular Signature in the Skin Preceding Long-Lasting T Cell Responses after Electroporated DNA Vaccination. J Immunol. 2020;204(12):3375–88. 32385135 10.4049/jimmunol.1900517 PMC7276943

[65] Nold-Petry CA, Nold MF, Zepp JA, Kim SH, Voelkel NF, Dinarello CA. IL-32-dependent effects of IL-1beta on endothelial cell functions. Proc Natl Acad Sci U S A. 2009;106(10):3883–8. 19228941 10.1073/pnas.0813334106 PMC2656174

[66] Dolgachev V, Panicker S, Balijepalli S, McCandless LK, Yin Y, Swamy S, et al. Electroporation-mediated delivery of FER gene enhances innate immune response and improves survival in a murine model of pneumonia. Gene Ther. 2018;25(5):359–75. 29907877 10.1038/s41434-018-0022-y PMC6195832

[67] Met O, Eriksen J, Svane IM. Studies on mRNA electroporation of immature and mature dendritic cells: effects on their immunogenic potential. Mol Biotechnol. 2008;40(2):151–60. 18543130 10.1007/s12033-008-9071-6

[68] Schultheis K, Smith TRF, Kiosses WB, Kraynyak KA, Wong A, Oh J, et al. Delineating the Cellular Mechanisms Associated with Skin Electroporation. Hum Gene Ther Methods. 2018;29(4):177–88. 29953259 10.1089/hgtb.2017.105 PMC6421993

[69] Polajzer T, Jarm T, Miklavcic D. Analysis of damage-associated molecular pattern molecules due to electroporation of cells in vitro. Radiol Oncol. 2020;54(3):317–28. 32726295 10.2478/raon-2020-0047 PMC7409611

[70] Chen W, McCluskey J. Immunodominance and immunodomination: critical factors in developing effective CD8+ T-cell-based cancer vaccines. Adv Cancer Res. 2006;95:203–47. 16860659 10.1016/S0065-230X(06)95006-4

[71] Akram A, Inman RD. Immunodominance: a pivotal principle in host response to viral infections. Clin Immunol. 2012;143(2):99–115. 22391152 10.1016/j.clim.2012.01.015

[72] Angeletti D, Kosik I, Santos JJS, Yewdell WT, Boudreau CM, Mallajosyula VVA, et al. Outflanking immunodominance to target subdominant broadly neutralizing epitopes. Proc Natl Acad Sci U S A. 2019;116(27):13474–9. 31213541 10.1073/pnas.1816300116 PMC6612916

[73] Yarchoan M, Gane EJ, Marron TU, Perales-Linares R, Yan J, Cooch N, et al. Personalized neoantigen vaccine and pembrolizumab in advanced hepatocellular carcinoma: a phase 1/2 trial. Nat Med. 2024;30(4):1044–53. 38584166 10.1038/s41591-024-02894-y PMC11031401

[74] Baden LR, El Sahly HM, Essink B, Kotloff K, Frey S, Novak R, et al. Efficacy and Safety of the mRNA-1273 SARS-CoV-2 Vaccine. N Engl J Med. 2021;384(5):403–16. 33378609 10.1056/NEJMoa2035389 PMC7787219

[75] Rojas LA, Sethna Z, Soares KC, Olcese C, Pang N, Patterson E, et al. Personalized RNA neoantigen vaccines stimulate T cells in pancreatic cancer. Nature. 2023;618(7963):144–50. 37165196 10.1038/s41586-023-06063-y PMC10171177

[76] Weber JS, Carlino MS, Khattak A, Meniawy T, Ansstas G, Taylor MH, et al. Individualised neoantigen therapy mRNA-4157 (V940) plus pembrolizumab versus pembrolizumab monotherapy in resected melanoma (KEYNOTE-942): a randomised, phase 2b study. Lancet. 2024;403(10427):632–44. 38246194 10.1016/S0140-6736(23)02268-7

[77] Hou X, Zaks T, Langer R, Dong Y. Lipid nanoparticles for mRNA delivery. Nat Rev Mater. 2021;6(12):1078–94. 34394960 10.1038/s41578-021-00358-0 PMC8353930

