# Supplementary material for: Improved efficacy of therapeutic HPV DNA vaccine using intramuscular injection with electroporation compared to conventional needle and needle-free jet injector methods
Source: Cell Biosci. 2024 Dec 25;14:154. doi: 10.1186/s13578-024-01338-x (PMC11670459; doi:10.1186/s13578-024-01338-x)
Supplement: Supplementary file 1 — Supplementary Material 1 [file 13578_2024_1338_MOESM1_ESM.pptx]

## Slide 1
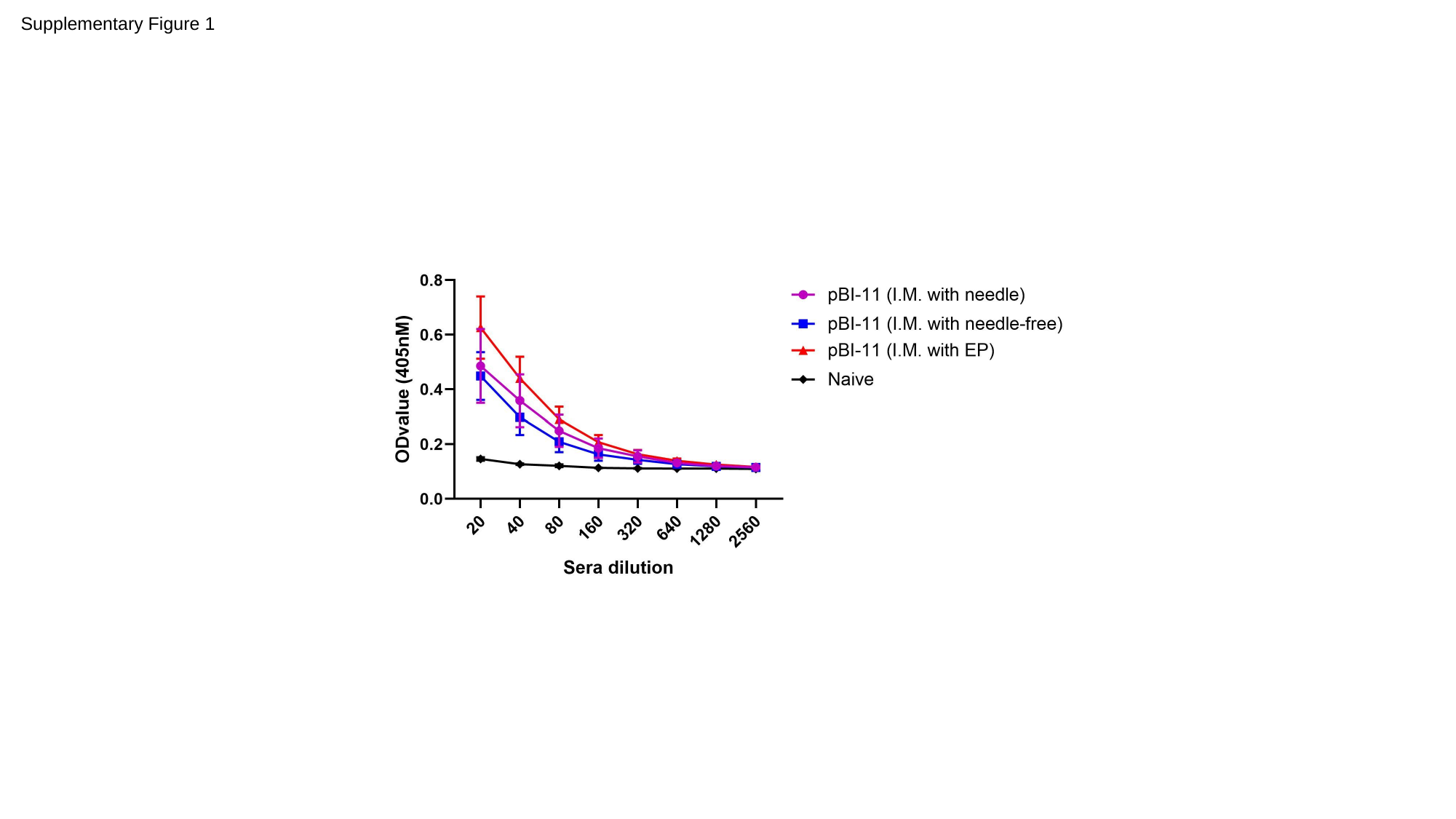

Supplementary Figure 1

## Slide 2
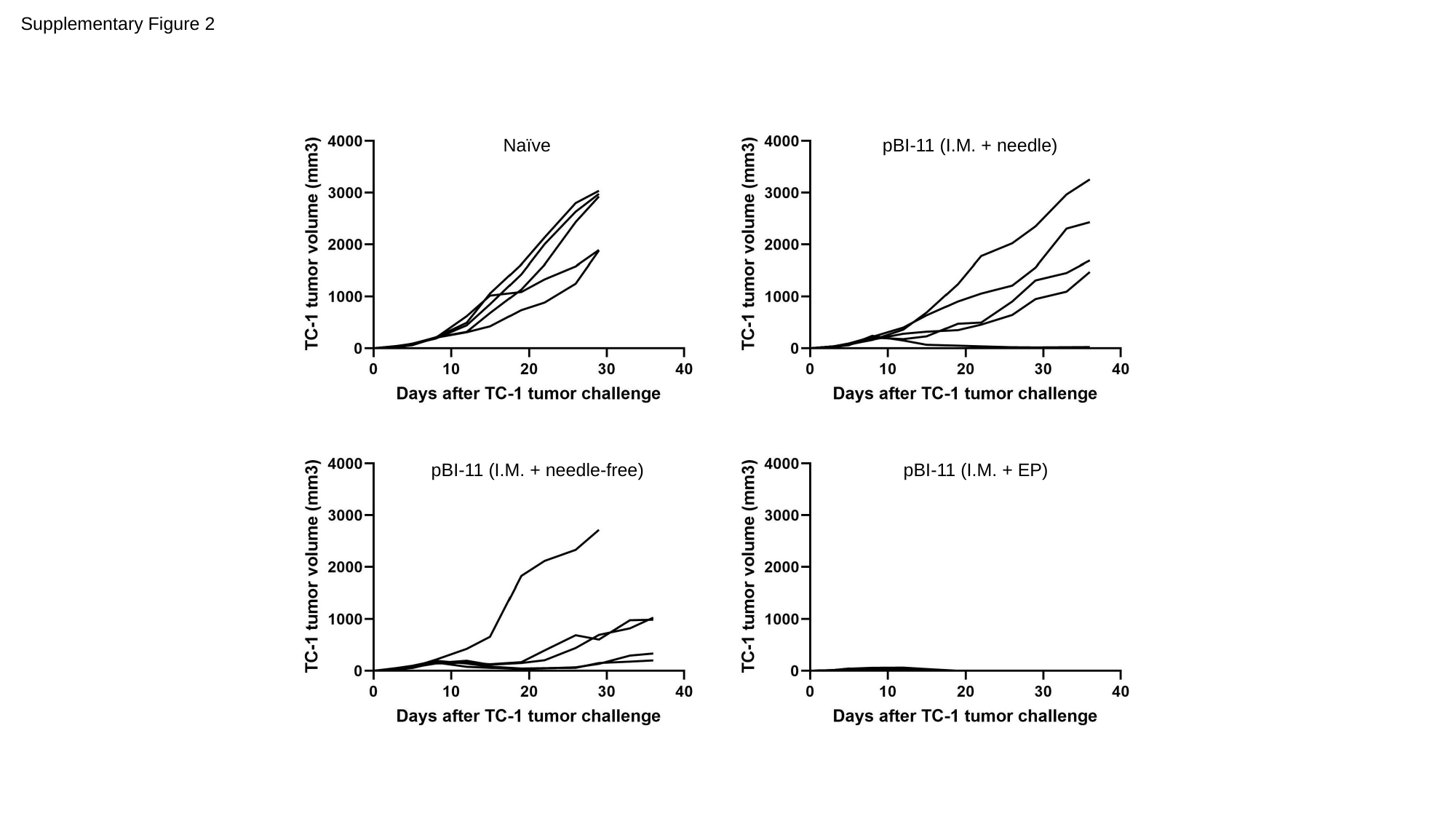

Supplementary Figure 2
Naïve
pBI-11 (I.M. + needle)
pBI-11 (I.M. + needle-free)
pBI-11 (I.M. + EP)

## Slide 3
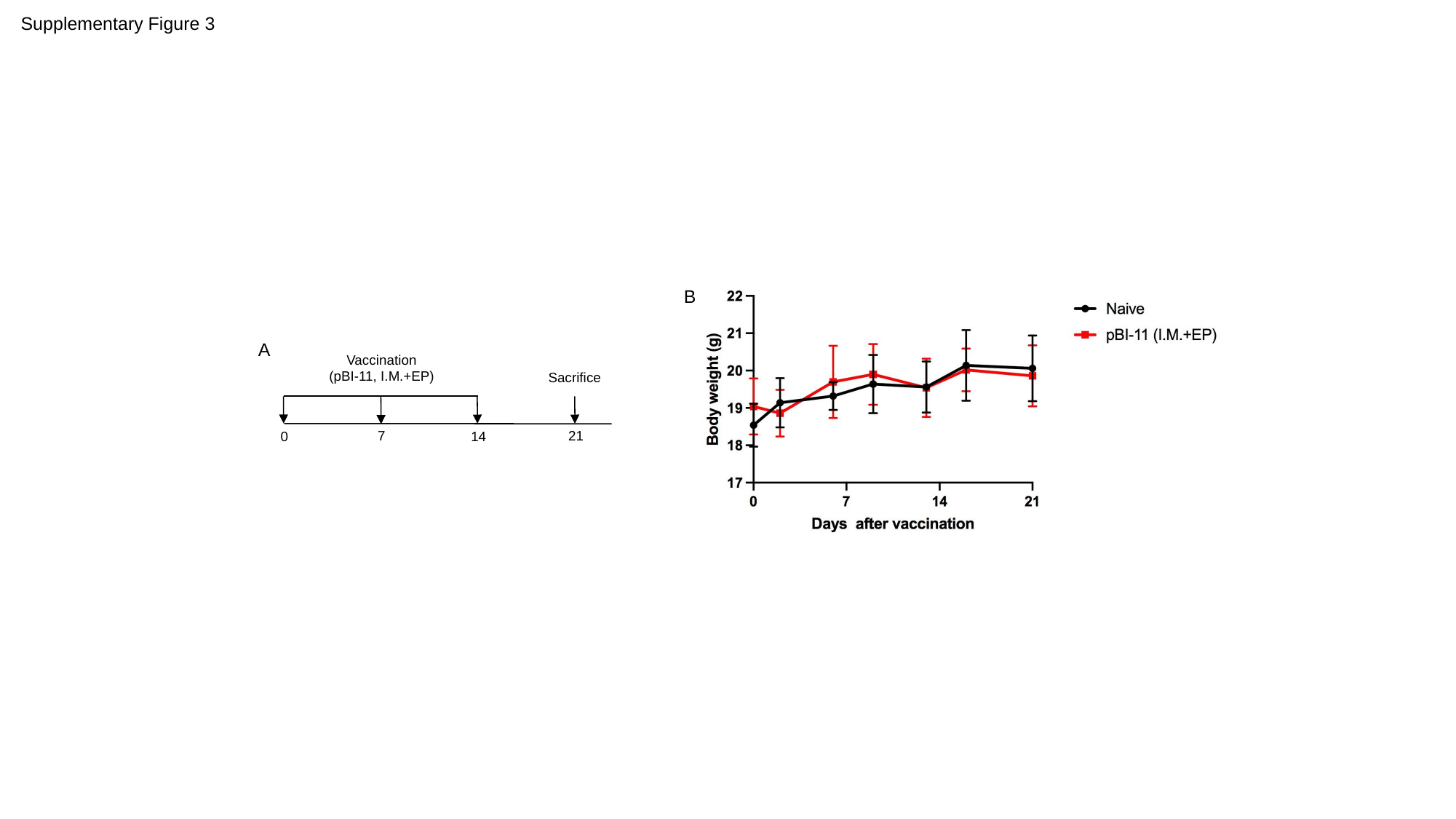

Supplementary Figure 3
B
A
Vaccination
(pBI-11, I.M.+EP)
Sacrifice
7
21
0
14

## Slide 4
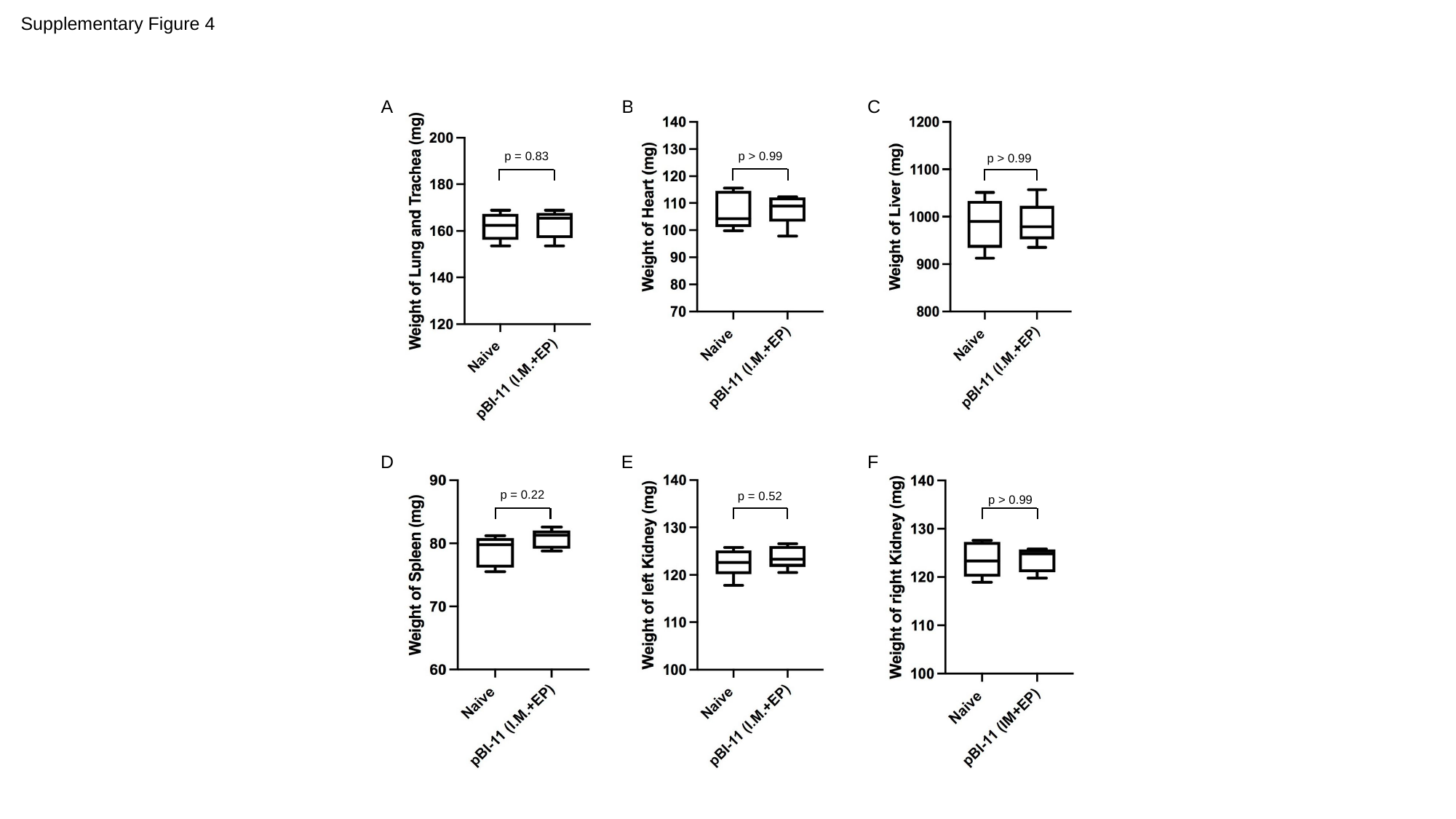

Supplementary Figure 4
B
p > 0.99
C
A
p = 0.83
p > 0.99
D
p = 0.22
E
p = 0.52
F
p > 0.99

## Slide 5
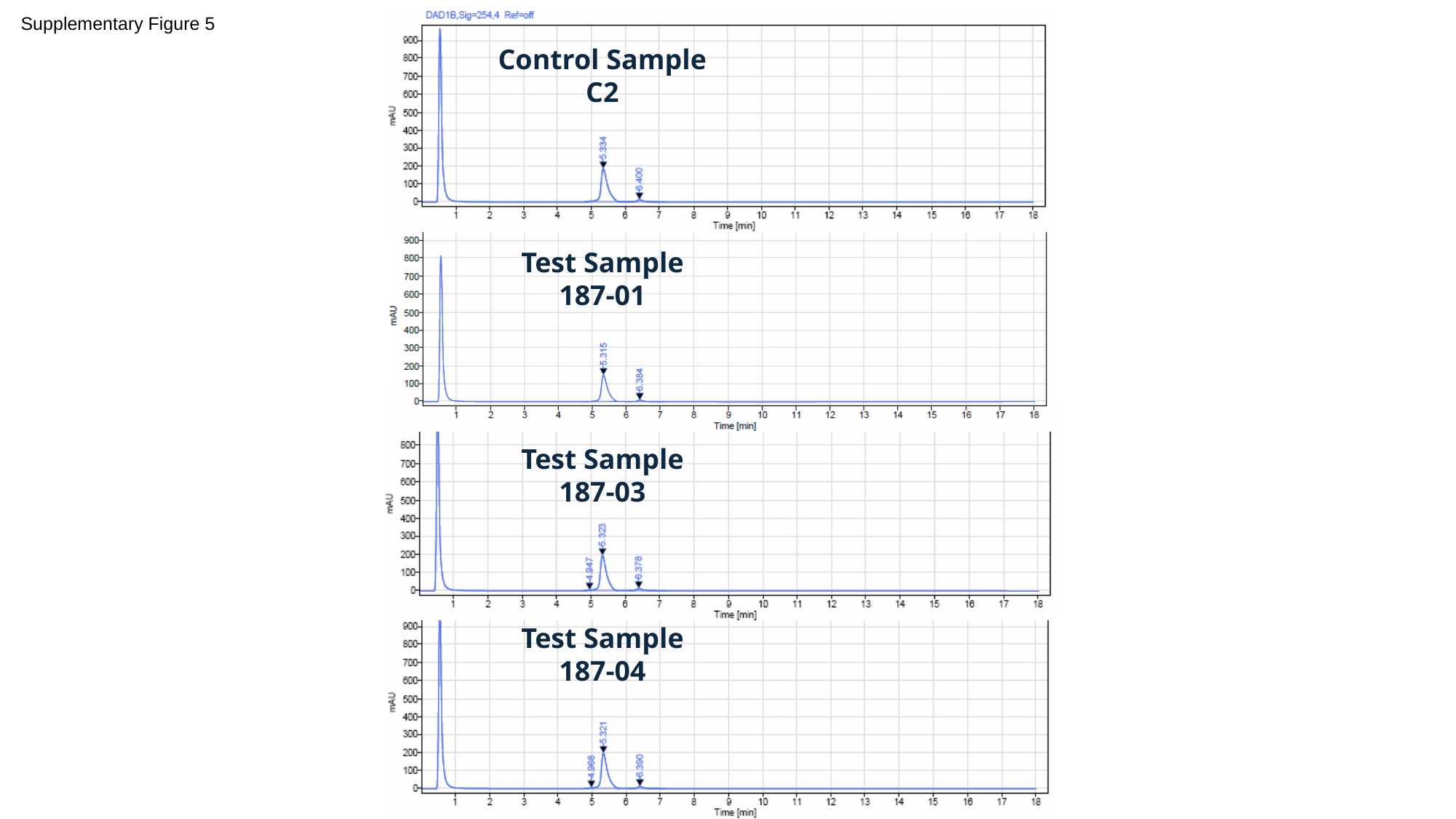

Supplementary Figure 5
Control Sample
C2
Test Sample
187-01
Test Sample
187-03
Test Sample
187-04

## Slide 6
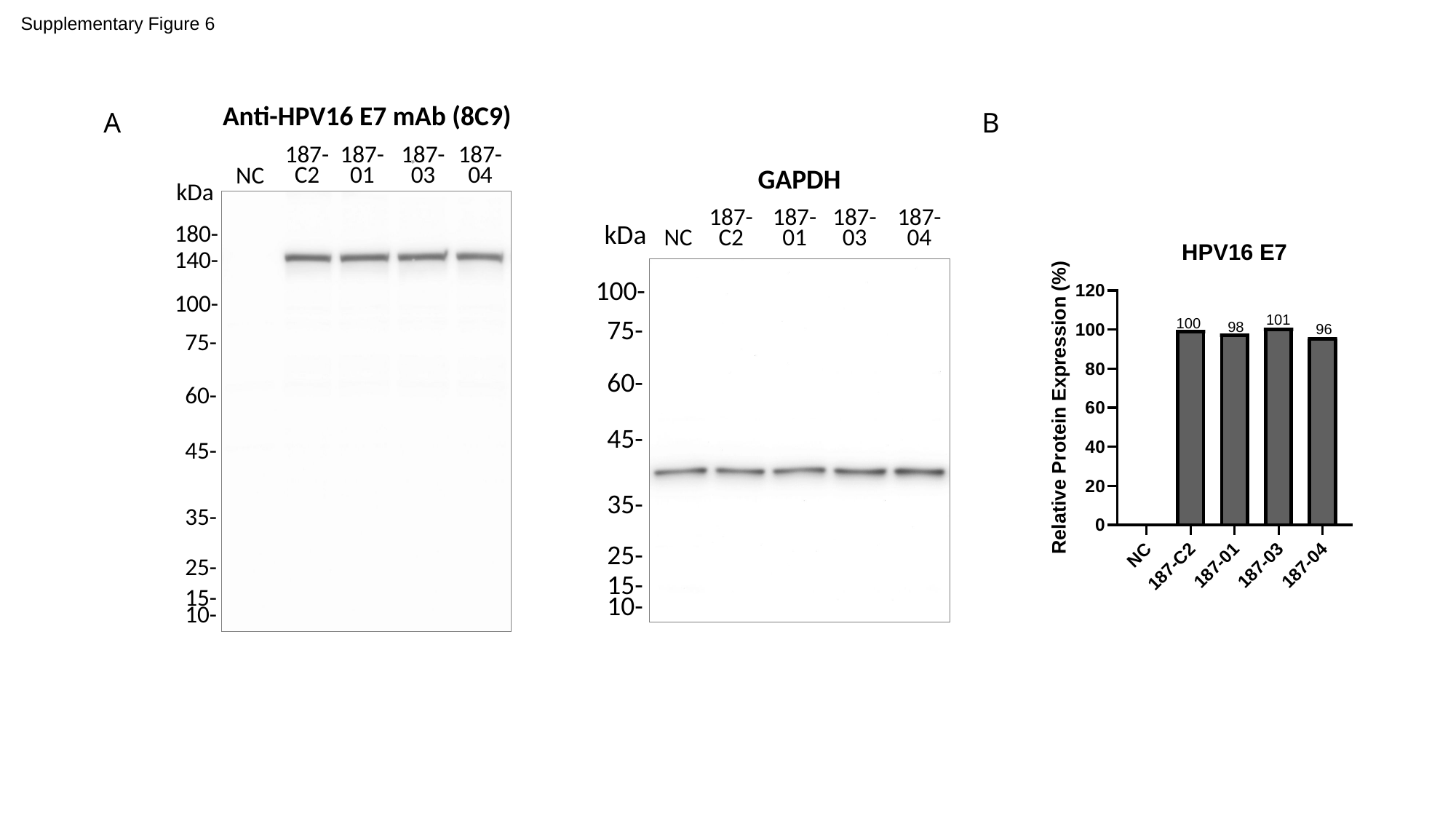

Supplementary Figure 6
Anti-HPV16 E7 mAb (8C9)
A
B
187-C2
187-01
187-03
187-04
GAPDH
NC
kDa
187-C2
187-01
187-03
187-04
kDa
180-
NC
140-
100-
100-
75-
75-
60-
60-
45-
45-
35-
35-
25-
25-
15-
15-
10-
10-
