# Supplementary material for: Improved efficacy of therapeutic HPV DNA vaccine using intramuscular injection with electroporation compared to conventional needle and needle-free jet injector methods
Source: Cell Biosci. 2024 Dec 25;14:154. doi: 10.1186/s13578-024-01338-x (PMC11670459; doi:10.1186/s13578-024-01338-x)

**Table S1**: Behavioral phenotype of mice receiving PBS or pBI-11 vaccination treatment delivered by TriGrid electroporation device. All mice received vaccine injections with either PBS (naïve) or pBI-11 DNA vaccination delivered by TriGrid electroporation device (treatment). N (no) and 2 represent normal behaviors. Numerical rating scale 0-3.

Assessment of abnormal gait in vaccinated mice

| Vaccination Group | Vaccination Mouse Number | Observation Date 1/17/24 | Observation Date  1/22/24 | Observation Date  1/27/24 | Observation Date  2/1/24 | Observation Date  2/6/24 |
| --- | --- | --- | --- | --- | --- | --- |
| Naïve | N-1 | N | N | N | N | N |
|  | N-2 | N | N | N | N | N |
|  | N-3 | N | N | N | N | N |
|  | N-4 | N | N | N | N | N |
|  | N-5 | N | N | N | N | N |
| pBI-11 | T-1 | N | N | N | N | N |
|  | T-2 | N | N | N | N | N |
|  | T-3 | N | N | N | N | N |
|  | T-4 | N | N | N | N | N |
|  | T-5 | N | N | N | N | N |

N represents typical behavior.

Assessment of abnormal posture in vaccinated mice

| Vaccination Group | Vaccination Mouse Number | Observation Date 1/17/24 | Observation Date  1/22/24 | Observation Date  1/27/24 | Observation Date  2/1/24 | Observation Date  2/6/24 |
| --- | --- | --- | --- | --- | --- | --- |
| Naïve | N-1 | N | N | N | N | N |
|  | N-2 | N | N | N | N | N |
|  | N-3 | N | N | N | N | N |
|  | N-4 | N | N | N | N | N |
|  | N-5 | N | N | N | N | N |
| pBI-11 | T-1 | N | N | N | N | N |
|  | T-2 | N | N | N | N | N |
|  | T-3 | N | N | N | N | N |
|  | T-4 | N | N | N | N | N |
|  | T-5 | N | N | N | N | N |

N represents typical behavior.

Assessment of freezing behavior

| Vaccination Group | Vaccination Mouse Number | Observation Date 1/17/24 | Observation Date  1/22/24 | Observation Date  1/27/24 | Observation Date  2/1/24 | Observation Date  2/6/24 |
| --- | --- | --- | --- | --- | --- | --- |
| Naïve | N-1 | N | N | N | N | N |
|  | N-2 | N | N | N | N | N |
|  | N-3 | N | N | N | N | N |
|  | N-4 | N | N | N | N | N |
|  | N-5 | N | N | N | N | N |
| pBI-11 | T-1 | N | N | N | N | N |
|  | T-2 | N | N | N | N | N |
|  | T-3 | N | N | N | N | N |
|  | T-4 | N | N | N | N | N |
|  | T-5 | N | N | N | N | N |

N represents typical behavior.

Wild Running

| Vaccination Group | Vaccination Mouse Number | Observation Date 1/17/24 | Observation Date  1/22/24 | Observation Date  1/27/24 | Observation Date  2/1/24 | Observation Date  2/6/24 |
| --- | --- | --- | --- | --- | --- | --- |
| Naïve | N-1 | N | N | N | N | N |
|  | N-2 | N | N | N | N | N |
|  | N-3 | N | N | N | N | N |
|  | N-4 | N | N | N | N | N |
|  | N-5 | N | N | N | N | N |
| pBI-11 | T-1 | N | N | N | N | N |
|  | T-2 | N | N | N | N | N |
|  | T-3 | N | N | N | N | N |
|  | T-4 | N | N | N | N | N |
|  | T-5 | N | N | N | N | N |

N represents typical behavior.

Stereotypes

| Vaccination Group | Vaccination Mouse Number | Observation Date 1/17/24 | Observation Date  1/22/24 | Observation Date  1/27/24 | Observation Date  2/1/24 | Observation Date  2/6/24 |
| --- | --- | --- | --- | --- | --- | --- |
| Naïve | N-1 | N | N | N | N | N |
|  | N-2 | N | N | N | N | N |
|  | N-3 | N | N | N | N | N |
|  | N-4 | N | N | N | N | N |
|  | N-5 | N | N | N | N | N |
| pBI-11 | T-1 | N | N | N | N | N |
|  | T-2 | N | N | N | N | N |
|  | T-3 | N | N | N | N | N |
|  | T-4 | N | N | N | N | N |
|  | T-5 | N | N | N | N | N |

N represents typical behavior.

Escape

| Vaccination Group | Vaccination Mouse Number | Observation Date 1/17/24 | Observation Date  1/22/24 | Observation Date  1/27/24 | Observation Date  2/1/24 | Observation Date  2/6/24 |
| --- | --- | --- | --- | --- | --- | --- |
| Naïve | N-1 | N | N | N | N | N |
|  | N-2 | N | N | N | N | N |
|  | N-3 | N | N | N | N | N |
|  | N-4 | N | N | N | N | N |
|  | N-5 | N | N | N | N | N |
| pBI-11 | T-1 | N | N | N | N | N |
|  | T-2 | N | N | N | N | N |
|  | T-3 | N | N | N | N | N |
|  | T-4 | N | N | N | N | N |
|  | T-5 | N | N | N | N | N |

N represents typical behavior.

Exploring

| Vaccination Group | Vaccination Mouse Number | Observation Date 1/17/24 | Observation Date  1/22/24 | Observation Date  1/27/24 | Observation Date  2/1/24 | Observation Date  2/6/24 |
| --- | --- | --- | --- | --- | --- | --- |
| Naïve | N-1 | 2 | 2 | 2 | 2 | 2 |
|  | N-2 | 2 | 2 | 2 | 2 | 2 |
|  | N-3 | 2 | 2 | 2 | 2 | 2 |
|  | N-4 | 2 | 2 | 2 | 2 | 2 |
|  | N-5 | 2 | 2 | 2 | 2 | 2 |
| pBI-11 | T-1 | 2 | 2 | 2 | 2 | 2 |
|  | T-2 | 2 | 2 | 2 | 2 | 2 |
|  | T-3 | 2 | 2 | 2 | 2 | 2 |
|  | T-4 | 2 | 2 | 2 | 2 | 2 |
|  | T-5 | 2 | 2 | 2 | 2 | 2 |

2 represents normal behavior.

Digging

| Vaccination Group | Vaccination Mouse Number | Observation Date 1/17/24 | Observation Date  1/22/24 | Observation Date  1/27/24 | Observation Date  2/1/24 | Observation Date  2/6/24 |
| --- | --- | --- | --- | --- | --- | --- |
| Naïve | N-1 | 2 | 2 | 2 | 2 | 2 |
|  | N-2 | 2 | 2 | 2 | 2 | 2 |
|  | N-3 | 2 | 2 | 2 | 2 | 2 |
|  | N-4 | 2 | 2 | 2 | 2 | 2 |
|  | N-5 | 2 | 2 | 2 | 2 | 2 |
| pBI-11 | T-1 | 2 | 2 | 2 | 2 | 2 |
|  | T-2 | 2 | 2 | 2 | 2 | 2 |
|  | T-3 | 2 | 2 | 2 | 2 | 2 |
|  | T-4 | 2 | 2 | 2 | 2 | 2 |
|  | T-5 | 2 | 2 | 2 | 2 | 2 |

2 represents normal behavior.

Grooming

| Vaccination Group | Vaccination Mouse Number | Observation Date 1/17/24 | Observation Date  1/22/24 | Observation Date  1/27/24 | Observation Date  2/1/24 | Observation Date  2/6/24 |
| --- | --- | --- | --- | --- | --- | --- |
| Naïve | N-1 | 2 | 2 | 2 | 2 | 2 |
|  | N-2 | 2 | 2 | 2 | 2 | 2 |
|  | N-3 | 2 | 2 | 2 | 2 | 2 |
|  | N-4 | 2 | 2 | 2 | 2 | 2 |
|  | N-5 | 2 | 2 | 2 | 2 | 2 |
| pBI-11 | T-1 | 2 | 2 | 2 | 2 | 2 |
|  | T-2 | 2 | 2 | 2 | 2 | 2 |
|  | T-3 | 2 | 2 | 2 | 2 | 2 |
|  | T-4 | 2 | 2 | 2 | 2 | 2 |
|  | T-5 | 2 | 2 | 2 | 2 | 2 |

2 represents normal behavior.

Rearing

| Vaccination Group | Vaccination Mouse Number | Observation Date 1/17/24 | Observation Date  1/22/24 | Observation Date  1/27/24 | Observation Date  2/1/24 | Observation Date  2/6/24 |
| --- | --- | --- | --- | --- | --- | --- |
| Naïve | N-1 | 2 | 2 | 2 | 2 | 2 |
|  | N-2 | 2 | 2 | 2 | 2 | 2 |
|  | N-3 | 2 | 2 | 2 | 2 | 2 |
|  | N-4 | 2 | 2 | 2 | 2 | 2 |
|  | N-5 | 2 | 2 | 2 | 2 | 2 |
| pBI-11 | T-1 | 2 | 2 | 2 | 2 | 2 |
|  | T-2 | 2 | 2 | 2 | 2 | 2 |
|  | T-3 | 2 | 2 | 2 | 2 | 2 |
|  | T-4 | 2 | 2 | 2 | 2 | 2 |
|  | T-5 | 2 | 2 | 2 | 2 | 2 |

2 represents normal behavior.

**Table S2**: Assessment for eschar formation at the vaccinated site

|  |  | **Time after 1^st^ Vaccination** | | **Time after 2^nd^ Vaccination** | | **Time after 3^rd^ Vaccination** | |
| --- | --- | --- | --- | --- | --- | --- | --- |
| Vaccination Group | Vaccination Mouse Number | 2 hours | 24 hours | 2 hours | 24 hours | 2 hours | 24 hours |
| pBI-11 IM+EP | 1 | 0 | 0 | 0 | 0 | 0 | 0 |
|  | 2 | 0 | 0 | 0 | 0 | 0 | 0 |
|  | 3 | 0 | 0 | 0 | 0 | 0 | 0 |
|  | 4 | 0 | 0 | 0 | 0 | 0 | 0 |
|  | 5 | 0 | 0 | 0 | 0 | 0 | 0 |

0- Within normal limits / No significant findings (unremarkable)

**Table S3**: Assessment of edema formation at vaccinated site

|  |  | **Time after 1^st^ Vaccination** | | **Time after 2^nd^ Vaccination** | | **Time after 3^rd^ Vaccination** | |
| --- | --- | --- | --- | --- | --- | --- | --- |
| Vaccination Group | Vaccination Mouse Number | 2 hours | 24 hours | 2 hours | 24 hours | 2 hours | 24 hours |
| pBI-11 IM+EP | 1 | 0 | 0 | 0 | 0 | 0 | 0 |
|  | 2 | 0 | 0 | 0 | 0 | 0 | 0 |
|  | 3 | 0 | 0 | 0 | 0 | 0 | 0 |
|  | 4 | 0 | 0 | 0 | 0 | 0 | 0 |
|  | 5 | 0 | 0 | 0 | 0 | 0 | 0 |

0 – Within normal limits / No significant findings (unremarkable)

**Table S4**: Complete blood count of vaccinated mice.

| Mouse Number | 1 | 2 | 3 | 4 | 5 | 6 | 7 | 8 | 9 | 10 |
| --- | --- | --- | --- | --- | --- | --- | --- | --- | --- | --- |
| Test Day | 2/7/2024 | 2/7/2024 | 2/7/2024 | 2/7/2024 | 2/7/2024 | 2/7/2024 | 2/7/2024 | 2/7/2024 | 2/7/2024 | 2/7/2024 |
| Species | Mouse | Mouse | Mouse | Mouse | Mouse | Mouse | Mouse | Mouse | Mouse | Mouse |
| Strain | C57BL/6 | C57BL/6 | C57BL/6 | C57BL/6 | C57BL/6 | C57BL/6 | C57BL/6 | C57BL/6 | C57BL/6 | C57BL/6 |
| Age (week) | 20 | 20 | 20 | 20 | 20 | 20 | 20 | 20 | 20 | 20 |
| Sex | Female | Female | Female | Female | Female | Female | Female | Female | Female | Female |
| Vaccination Group Number | T-1 | T-2 | T-3 | T-4 | T-5 | N-1 | N-2 | N-3 | N-4 | N-5 |
| RBC (M/uL) | 10.30 | 10.53 | 10.25 | 10.71 | 10.69 | 10.24 | 10.51 | 10.97 | 10.63 | 10.93 |
| HGB (g/dL) | 16.1 | 16.4 | 15.9 | 16.6 | 16.7 | 15.7 | 16.3 | 17.1 | 16.7 | 16.9 |
| HCT (%) | 49.6 | 51.4 | 49.1 | 51.5 | 51.4 | 48.4 | 50.1 | 52.8 | 51.4 | 52.1 |
| MCV (fL) | 48.2 | 48.8 | 47.9 | 48.2 | 48.1 | 47.3 | 47.7 | 48.1 | 48.4 | 47.7 |
| MCH (pg) | 15.6 | 15.4 | 15.5 | 15.5 | 15.6 | 15.3 | 15.5 | 15.6 | 15.7 | 15.5 |
| MCHC (g/dL) | 32.5 | 31.9 | 32.4 | 32.2 | 32.5 | 32.4 | 32.5 | 32.4 | 32.5 | 32.4 |
| RDW-SD (fL) | 28.5 | 30.0 | 27.6 | 28.9 | 27.9 | 28.2 | 26.6 | 27.6 | 27.5 | 27.8 |
| RDW-CV (%) | 24.1 | 24.6 | 23.3 | 24.4 | 24.1 | 24.2 | 23.4 | 23.9 | 22.5 | 24.3 |
| RET (K/ul) | 398.6 | 299.1 | 279.3 | 336.3 | 303.6 | 345.1 | 324.8 | 337.9 | 380.6 | 334.5 |
| IRF (%) | 56.8 | 51.4 | 51.4 | 53.2 | 48.9 | 51.9 | 48.8 | 53.1 | 53.9 | 47.0 |
| LFR (%) | 43.2 | 48.6 | 48.6 | 46.8 | 51.1 | 48.1 | 51.2 | 46.9 | 46.1 | 53.0 |
| MFR (%) | 19.4 | 18.7 | 19.2 | 17.7 | 19.6 | 19.2 | 18.9 | 20.8 | 20.0 | 20.5 |
| HFR (%) | 37.4 | 34.8 | 32.2 | 35.5 | 29.3 | 32.7 | 29.9 | 32.3 | 33.9 | 26.5 |
| RET-He (pg) | 17.2 | 17.3 | 16.5 | 17.4 | 16.7 | 16.7 | 16.6 | 16.7 | 17.1 | 16.4 |
| PLT (K/uL) | 914 | 848 | 915 | 958 | 950 | 951 | 942 | 1018 | 1002 | 946 |
| PDW (fL) | 6.6 | 6.5 | 6.5 | 6.7 | 6.7 | 6.4 | 6.5 | 6.7 | 7.0 | 6.6 |
| MPV (fL) | 7.9 | 7.9 | 7.8 | 7.8 | 7.8 | 7.8 | 7.8 | 7.7 | 7.7 | 7.7 |
| P-LCR (%) | 1.9 | 4.3 | 1.6 | 3.3 | 1.7 | 1.8 | 2.0 | 2.4 | 2.0 | 2.4 |
| PCT (%) | 0.72 | 0.67 | 0.71 | 0.75 | 0.74 | 0.74 | 0.73 | 0.78 | 0.77 | 0.73 |
| WBC (K/uL) | 9.82 | 9.91 | 9.09 | 9.54 | 8.16 | 11.03 | 8.86 | 11.28 | 9.83 | 8.65 |
| NEUT (K/uL) | 1.04 | 1.03 | 0.89 | 0.73 | 0.73 | 1.02 | 0.88 | 1.08 | 0.81 | 0.49 |
| NEUT (%) | 10.6 | 10.4 | 9.9 | 7.6 | 9.0 | 9.2 | 9.9 | 9.6 | 8.3 | 5.8 |
| LYMPH (K/uL) | 8.55 | 8.65 | 8.02 | 8.65 | 7.29 | 9.79 | 7.76 | 9.95 | 8.89 | 8.04 |
| LYMPH (%) | 87.1 | 87.3 | 88.2 | 90.7 | 89.3 | 88.8 | 87.6 | 88.2 | 90.4 | 92.9 |
| MONO (K/uL) | 0.01 | 0.06 | 0.04 | 0.03 | 0.04 | 0.05 | 0.02 | 0.02 | 0.01 | 0.01 |
| MONO (%) | 0.1 | 0.6 | 0.4 | 0.3 | 0.5 | 0.5 | 0.2 | 0.2 | 0.1 | 0.1 |
| EO (K/uL) | 0.22 | 0.17 | 0.14 | 0.13 | 0.09 | 0.17 | 0.20 | 0.23 | 0.11 | 0.09 |
| EO (%) | 2.2 | 1.7 | 1.5 | 1.4 | 1.1 | 1.5 | 2.3 | 2.0 | 1.1 | 1.0 |
| BASO (K/uL) | 0.00 | 0.00 | 0.00 | 0.00 | 0.01 | 0.00 | 0.00 | 0.00 | 0.01 | 0.02 |
| BASO (%) | 0.0 | 0.0 | 0.0 | 0.0 | 0.1 | 0.0 | 0.0 | 0.0 | 0.1 | 0.2 |

Summary of complete blood count studies in vaccinated mice using serum one week after final vaccination

Abbreviations: RBC = Red Blood Cell Count; HGB = Hemoglobin value; HCT = Hematocrit value; MCV = Mean Corpuscular Volume; MHC = Mean Corpuscular Hemoglobin; MCHC = Mean Corpuscular Hemoglobin Concentration; RDW-SD = Red Cell Distribution Width Standard Deviation; RDW-CV = Red Cell Distribution Width Coefficient of Variation; RET = Reticulocytes; IRF =Immature Reticulocyte Fraction; LFR = Low Fluorescence Ratio; MFR = Medium Fluorescence Ratio; HFR = High Fluorescence Ratio; RET-He = Retic Hemoglobin; PLT = Platelet count; PDW = Platelet Distribution Width; MPV = Mean Platelet Volume ; P-LCR = Platelet Large Cell Ratio; PCT = Plateletcrit value; WBC = White Blood Cell Count; NEUT = Neutrophil percent; LYMPH = Lymphocyte percent; MONO = Monocyte; EO = Eosinophil; BASO = Basophil

**Table S5**: Summary of the biochemistry study in vaccinated mice

| Mouse Number | 1 | 2 | 3 | 4 | 5 | 6 | 7 | 8 | 9 | 10 |
| --- | --- | --- | --- | --- | --- | --- | --- | --- | --- | --- |
| Test Day | 2/7/2023 | 2/7/2023 | 2/7/2023 | 2/7/2023 | 2/7/2023 | 2/7/2023 | 2/7/2023 | 2/7/2023 | 2/7/2023 | 2/7/2023 |
| Species | Mouse | Mouse | Mouse | Mouse | Mouse | Mouse | Mouse | Mouse | Mouse | Mouse |
| Strain | C57BL/6 | C57BL/6 | C57BL/6 | C57BL/6 | C57BL/6 | C57BL/6 | C57BL/6 | C57BL/6 | C57BL/6 | C57BL/6 |
| Age (week) | 20 | 20 | 20 | 20 | 20 | 20 | 20 | 20 | 20 | 20 |
| Sex | Female | Female | Female | Female | Female | Female | Female | Female | Female | Female |
| Vaccination Group Number | T-1 | T-2 | T-3 | T-4 | T-5 | N-1 | N-2 | N-3 | N-4 | N-5 |
| ALP (U/L) | 111 | 101 | 115 | 127 | 128 | 121 | 125 | 115 | 123 | 127 |
| AST (U/L) | 40 | 71 | 55 | 63 | 55 | 48 | 46 | 46 | 62 | 51 |
| ALT (U/L) | 23 | 28 | 24 | 26 | 25 | 27 | 30 | 24 | 29 | 25 |
| CK (U/L) | 70 | 88 | 189 | 139 | 149 | 89 | 148 | 116 | 124 | 166 |
| ALB (g/dL) | 3.2 | 3.6 | 3.4 | 3.6 | 3.5 | 3.2 | 3.1 | 3.1 | 3.5 | 3.2 |
| TBIL (mg/dL) | 0.2 | 0.4 | 0.2 | 0.3 | 0.2 | 0.2 | 0.2 | 0.2 | 0.3 | 0.2 |
| TP (g/dL) | 5.0 | 5.6 | 5.4 | 5.5 | 5.5 | 5.1 | 4.9 | 4.9 | 5.4 | 5.1 |
| GLOBULIN (g/dL) | 1.8 | 2.0 | 2.0 | 1.9 | 2.0 | 1.9 | 1.8 | 1.8 | 1.9 | 1.9 |
| BIL-con (mg/dL) | 0.0 | 0.0 | 0.0 | 0.0 | 0.0 | 0.0 | 0.0 | 0.0 | 0.0 | 0.0 |
| BUN (mg/dL) | 20 | 25 | 21 | 28 | 25 | 24 | 27 | 22 | 25 | 23 |
| CREA (mg/dL) | 0.2 | 0.2 | 0.2 | 0.2 | 0.1 | 0.2 | 0.2 | 0.2 | 0.2 | 0.2 |
| CHOL (mg/dL) | 92 | 110 | 110 | 108 | 108 | 91 | 84 | 99 | 100 | 94 |
| GLUC (mg/dL) | 147 | 113 | 138 | 119 | 122 | 136 | 144 | 143 | 133 | 125 |
| CA (mg/dL) | 8.6 | 8.4 | 9.0 | 8.8 | 9.0 | 8.6 | 8.9 | 8.9 | 8.8 | 8.7 |
| PHOS (mg/dL) | 5.8 | 6.2 | 5.9 | 6.5 | 6.3 | 5.7 | 5.9 | 5.6 | 5.7 | 5.6 |
| TCO2 (mmol/L) | 16 | 13 | 14 | 13 | 16 | 13 | 15 | 14 | 12 | 13 |
| CL (mmol/L) | 108 | 113 | 110 | 112 | 110 | 108 | 109 | 111 | 108 | 111 |
| K (mmol/L) | 4.7 | 7.4 | 5.9 | 6.4 | 6.0 | 5.3 | 5.2 | 5.4 | 5.6 | 5.1 |
| ALB/GLOB ratio | 1.8 | 1.8 | 1.7 | 1.9 | 1.8 | 1.7 | 1.7 | 1.7 | 1.8 | 1.7 |
| NA (mmol/L) | 151 | 156 | 154 | 156 | 154 | 151 | 151 | 154 | 154 | 155 |
| BUN/CREA ratio | 100.0 | 125.0 | 105.0 | 140.0 | 250.0 | 120.0 | 135.0 | 110.0 | 125.0 | 115.0 |
| BIL-uncon (mg/dL) | 0.2 | 0.4 | 0.2 | 0.3 | 0.2 | 0.2 | 0.2 | 0.2 | 0.3 | 0.2 |
| NA/K ratio | 32 | 21 | 26 | 24 | 26 | 28 | 29 | 29 | 28 | 30 |

Summary of biochemistry study in vaccinated mice using serum one week after final vaccination

Abbreviations: ALB = Albumin; ALB/GLOB = Albumin to Globulin; ALP = Alkaline phosphatase; ALT = Alanine Aminotransferase; AST = Aspartate Aminotransferase; BIL-con = Bilirubin conjugated; BIL-uncon = Bilirubin conjugated; BUN = Blood Urea Nitrogen; BUN/CREA = Blood Urea Nitrogen to Creatinine; CA = Calcium; CHOL = Cholesterol; CK = Creatine Kinase; CL = Chloride; CREA = Creatinine; GLUC = Glucose; K = Potassium; NA = Sodium; TBIL = Total Bilrubin; TCO2 = bicarbonate TCO2; TP = Total Protein;

**Table S6** Histological examination of key organs in mice receiving vaccination.

| Vaccination Group | pBI-11 (IM+EP) | Control |
| --- | --- | --- |
| Date | 02/07/2024 | 02/07/2024 |
| Brain | WNL/NSF | WNL/NSF |
| Thymus | WNL/NSF | WNL/NSF |
| Trachea-Lung | WNL/NSF | WNL/NSF |
| Heart | WNL/NSF | WNL/NSF |
| Stomach | WNL/NSF | WNL/NSF |
| Small intestine | WNL/NSF | WNL/NSF |
| Large intestine | WNL/NSF | WNL/NSF |
| Pancreas | WNL/NSF | WNL/NSF |
| Liver | WNL/NSF | WNL/NSF |
| Right kidney | WNL/NSF | WNL/NSF |
| Left kidney | WNL/NSF | WNL/NSF |
| Ovaries | WNL/NSF | WNL/NSF |
| Fallopian Tubes | WNL/NSF | WNL/NSF |
| Uterus | WNL/NSF | WNL/NSF |
| Adrenal glands | WNL/NSF | WNL/NSF |
| Spleen | WNL/NSF | WNL/NSF |

WNL/NSF = Within normal limits / No significant findings (unremarkable)

**Table S7** Analytical tests, acceptance specifications, and testing results for device compatibility.


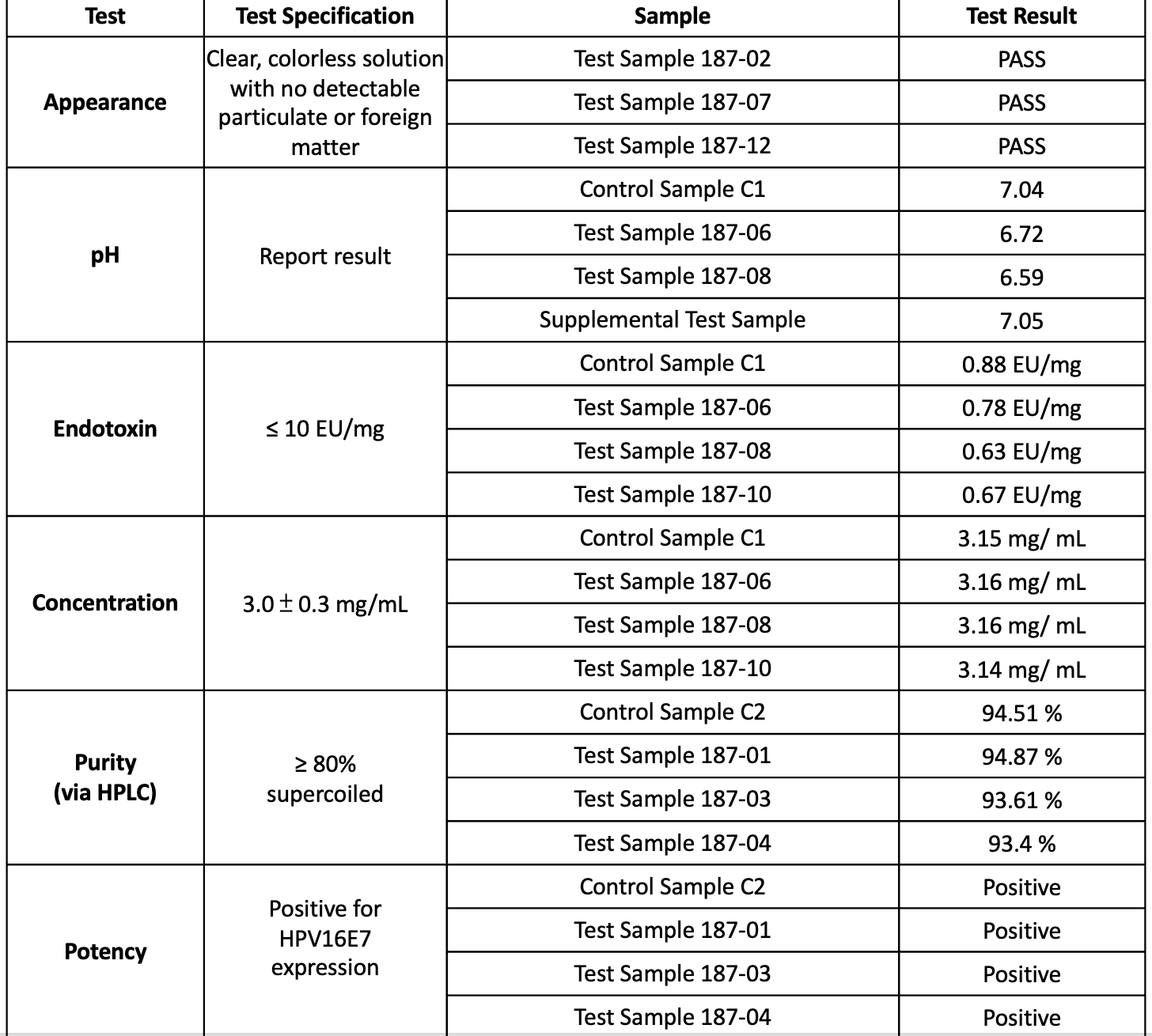

Supplement: Supplementary file 2 — Supplementary Material 2 [file 13578_2024_1338_MOESM2_ESM.docx]
